# Evaluation of Mechanical and Durability Performance of Concrete with and Without Surface-Treated Plastic Fine Aggregates

**DOI:** 10.3390/ma19173602

**Published:** 2026-08-25

**Authors:** Siva Ikkurthi, Qingli Dai

**Affiliations:** Civil, Environmental, and Geospatial Engineering, Michigan Technological University, 6 Houghton, 1400 Townsend Drive, Houghton, MI 49931, USA

**Keywords:** plastic aggregate concrete, plastic waste materials, HDPE and PET, surface treatment, drying shrinkage, freeze–thaw

## Abstract

Global plastic waste generation and excessive sand extraction are major environmental challenges, but replacing fine aggregate with plastic waste often degrades concrete performance. This work characterizes concrete incorporating recycled HDPE and PET fine aggregates at a 10% volumetric replacement level, with and without polymer-specific surface treatment, across fresh, mechanical, and durability properties. Untreated plastic aggregate generally lowered mechanical performance due to low polymer stiffness, weak plastic–paste bonding, and greater interfacial void formation. Surface treatment partially offsets these effects by strengthening the plastic–paste bond. H_2_O_2_-treated HDPE granules recovered the 28-day elastic modulus to within 3% of the control while also improving compressive strength, ultrasonic pulse velocity, and freeze–thaw resistance. H_2_O_2_-treated HDPE chips showed the highest electrical resistivity and the lowest permeable void content. NaOH-treated PET chips gave the lowest chloride penetrability and the greatest drying shrinkage reduction, approximately 25% relative to the control, though NaOH produced no resistivity gain for PET-C. Freeze–thaw durability factor increased with surface treatment for HDPE-G and PET-C, with HDPE-G-T exhibiting the highest durability factor among the recycled plastic mixtures at 94.20%. These results show that surface-treated recycled HDPE and PET fine aggregate can be incorporated at 10% replacement while maintaining acceptable mechanical and durability performance, supporting recycled plastics as a viable partial fine-aggregate replacement.

## 1. Introduction

About 430.9 million tons of global plastic waste were generated in 2024, with high-density polyethylene (HDPE) and polyethylene terephthalate (PET) accounting for approximately 79 million tons, primarily from packaging applications [1]. Current disposal pathways direct 50% to landfills, 19% to incineration, and only 9% to recycling, while 22% enter unmanaged waste streams with significant environmental consequences [2,3]. These disposal pathways are linked to several environmental concerns, including soil degradation and reduced land fertility, air pollution from incineration, and the widespread dispersion of plastic debris into natural systems [4,5,6]. At the same time, the construction industry faces increasing scarcity of natural fine aggregates, as sand is not a renewable resource and too much extraction causes significant environmental degradation [6]. Using recycled plastic waste as a partial replacement for fine aggregate offers a high-volume reuse pathway that addresses both waste accumulation and resource depletion within a circular economy framework [7,8].

Replacing the natural fine aggregate with recycled plastic waste, particularly PET and HDPE, carries several advantages that have sustained research interest in this area. Because thermoplastic particles have a substantially lower specific gravity than mineral aggregates, incorporating plastic aggregates (PAs) can reduce concrete density and promote the development of lightweight concrete [7,8]. Previous studies have also shown that workability is strongly influenced by plastic particle morphology, surface texture, and replacement level. Smooth or rounded particles may improve flowability by reducing interparticle friction, whereas irregular or flat particles may reduce workability due to poor packing efficiency and increased particle interactions [9,10]. Despite these potential benefits, the PA typically reduces the concrete’s mechanical performance because thermoplastic particles have substantially lower stiffness and strength than natural mineral aggregates. In addition, the hydrophobic and chemically inert nature of plastic surfaces results in weak interfacial transition zones (ITZs) characterized by poor adhesion, increased interfacial porosity, and inefficient stress transfer between the cement matrix and the aggregate phase [11,12]. These interfacial deficiencies introduce microstructural discontinuities that weaken load transfer efficiency and consequently reduce compressive, tensile, and flexural strength, as well as the modulus of elasticity [13]. The extent of performance reduction depends strongly on polymer type, replacement level, particle geometry, and water-to-cement ratio [14]. Moderate replacement levels may still provide acceptable mechanical performance while improving ductility and energy absorption capacity [13].

Durability performance represents another critical challenge for PA concrete. Weak and porous ITZs may facilitate moisture ingress and ionic transport, thereby reducing resistance to chloride penetration and freeze–thaw deterioration [14,15]. Conversely, the non-absorptive and chemically stable nature of plastic particles can reduce water uptake and disrupt continuous transport pathways at controlled replacement levels [12,16]. Several studies have reported improved chloride resistance and reduced permeability in PA concrete systems because impermeable PA can disrupt interconnected transport pathways. HDPE aggregates have also demonstrated enhanced durability in chemically aggressive environments because of their high chemical stability and resistance to degradation [8,16]. These findings indicate that the combined influence of particle morphology, interfacial bonding, pore structure, and the continuity of ionic transport pathways controls the durability of concrete containing recycled PA.

The quality of the interfacial transition zone (ITZ) is a key factor controlling the mechanical response and durability of concrete incorporating recycled PA. Compared with conventional mineral aggregates, recycled plastic particles exhibit low surface energy and relatively smooth surfaces, which reduce cement paste adhesion and favor the formation of less dense interfacial transition zones [11]. These interfacial regions reduce stress transfer efficiency and facilitate moisture and chloride ingress through the concrete [14,17]. The extent of these effects depends on both particle morphology and surface condition, with angular or elongated particles generally producing less favorable interfacial characteristics than compact granular particles [13,14]. Surface treatment has been proposed to improve the compatibility of recycled plastic particles with cementitious matrices by increasing surface roughness and incorporating polar functional groups, thereby enhancing interfacial adhesion and partially offsetting the performance reductions associated with the use of plastic aggregate [18,19].

Although previous studies have demonstrated the potential benefits of surface treatment [18,20], several important limitations remain. Most investigations have examined HDPE and PET independently, limiting direct comparison of polymer-specific behavior under identical experimental conditions [7,21]. In addition, the influence of particle morphology, particularly granular versus chip geometries, has not been systematically isolated despite its importance in governing packing density, interfacial surface area, and transport behavior [7,22]. Furthermore, surface treatment effects are rarely evaluated concurrently across fresh properties, mechanical performance, durability, and transport characteristics, limiting the ability to establish direct processing–structure–property relationships [20,21].

This study addresses these gaps directly by evaluating how polymer type, particle morphology, and polymer-specific surface treatment jointly influence the mechanical and durability performance of plastic aggregate concrete. HDPE granules (HDPE-G), HDPE chips (HDPE-C), and PET chips (PET-C) each partially replaced natural fine aggregate at a fixed 10% volumetric level and were tested both with and without surface treatment. Mechanical performance was characterized through compressive strength, splitting tensile strength, flexural strength, modulus of elasticity, and ultrasonic pulse velocity measurements. At the same time, durability was assessed via rapid chloride penetrability, electrical resistivity, water absorption, permeable voids, freeze–thaw resistance, and drying shrinkage tests. By evaluating all these properties within a single controlled experimental framework, this study aims to establish direct relationships among polymer type, particle morphology, surface treatment, and concrete performance and provide mechanistic insight into how interfacial modification governs the behavior of plastic aggregate concrete.

## 2. Materials and Mix Design

### 2.1. Materials

Type IL Portland cement meeting ASTM C595 [23] specifications, with a specific gravity of 3.14 g/cm^3^ and Blaine fineness of 320 m^2^/kg, served as the binder throughout this study. Natural river sand conforming to ASTM C33 [24] grading requirements was used as the reference for the fine aggregate, with a specific gravity of 2.71 g/cm^3^, a fineness modulus of 2.71, and a dominant particle size fraction of 0.30–0.60 mm. Crushed granite coarse aggregate (CA) with a maximum size of 19 mm and a specific gravity of 2.65 g/cm^3^ was incorporated at constant proportions across all mixtures. Tap water meeting ASTM C1602 [25] requirements was used for mixing and curing operations.

**Figure 1:**
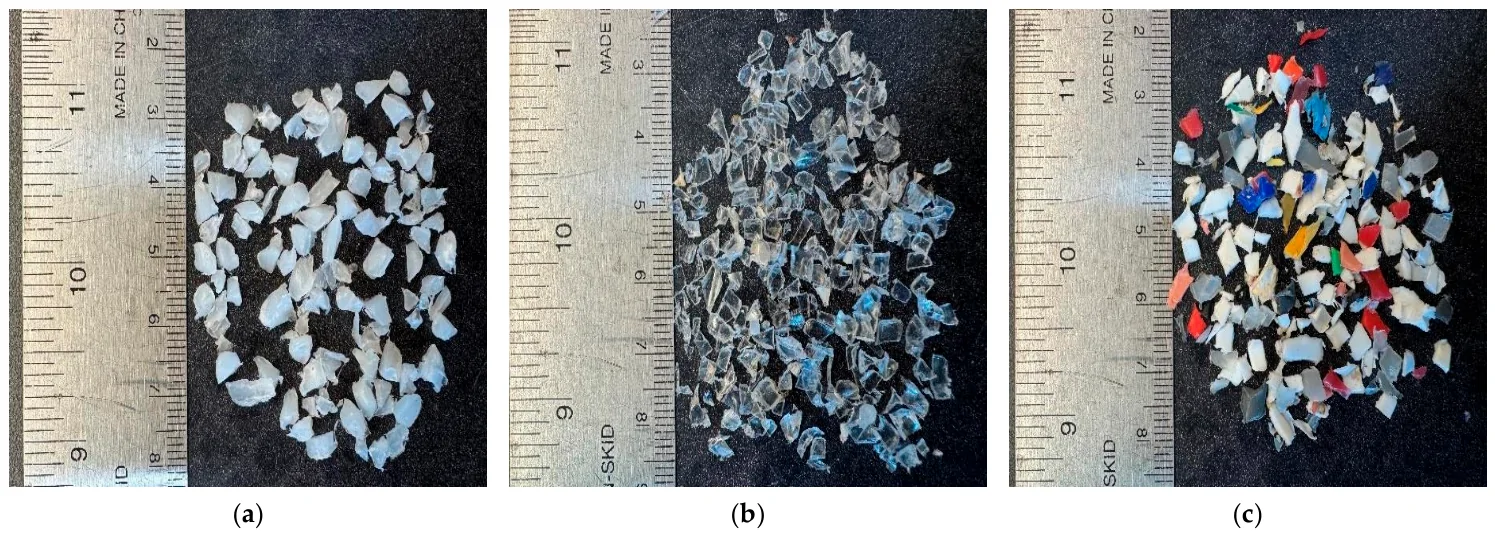
Processed plastic aggregates. (**a**) HDPE granules; (**b**) Clear PET chips; (**c**) Mixed post-consumer HDPE chips.

**Figure 2:**
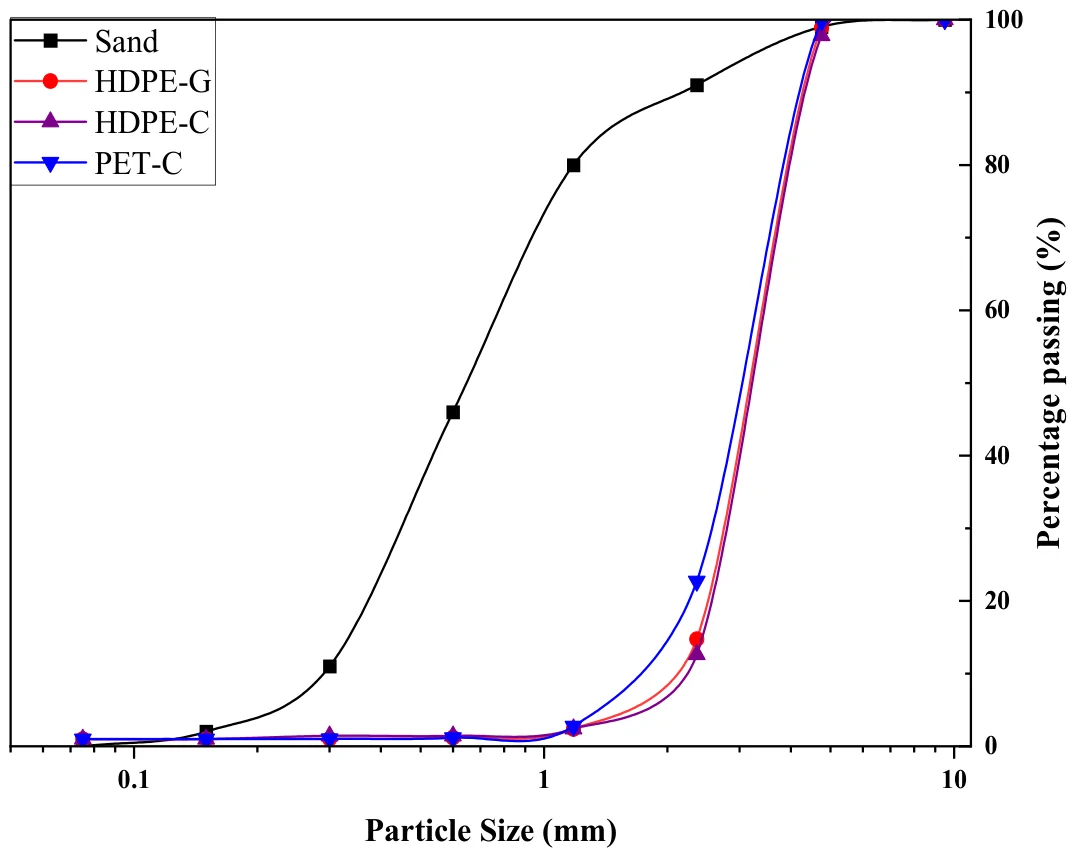
Particle size distributions of natural sand and recycled plastic aggregates.

Post-consumer HDPE packaging containers and PET beverage bottles were manually sorted, cleaned with water and a mild detergent, and air-dried prior to mechanical processing. HDPE-G, produced from post-consumer HDPE through industrial recycling processes, was supplied by Entech Inc. (Middlebury, IN, USA) [26]. HDPE-C and PET-C were produced using a cross-cut shredder (Allegheny 16-75CX, Delmont, PA, USA), with PET-C processed following cap and label removal. Feedstock was shredded at approximately 2 min/lb through progressively smaller screens (4.75, 2.36, and 1.18 mm) over three to four passes, with material retained on the 1.18 mm sieve collected as the final product (Table 1). All plastic particles were subsequently sieved in accordance with ASTM C136 [27] to obtain a uniform particle-size range of 2–4 mm (Figure 1). Consequently, nearly all plastic particles passed the 4.75 mm sieve, whereas only 11.35–21.93% and approximately 2% passed the 2.36 mm and 1.18 mm sieves, respectively, confirming that the recycled plastic aggregates (PAs) were predominantly coarse, sand-sized particles. The fineness moduli of HDPE-G, HDPE-C, and PET-C were 4.86, 4.90, and 4.76, respectively, compared with 2.71 for the natural sand, confirming that the recycled PA possessed a substantially coarser and narrower particle size distribution (PSD). Consequently, comparisons between the recycled plastic mixtures and the control may reflect the combined effects of polymer incorporation, aggregate gradation, particle packing, and interfacial behavior. However, because each untreated–treated pair was prepared from the same recycled plastic source and sieved to the identical 2–4 mm particle size range, comparisons within the same polymer system primarily isolate the influence of surface treatment under comparable gradation conditions. Because the specific gravities of HDPE and PET were 0.95 and 1.39 g/cm^3^, respectively, compared with 2.71 g/cm^3^ for natural sand, aggregate replacement was performed on an equal-volume basis to eliminate the influence of density differences. The material physical properties are summarized in Table 1, while the complete PSDs are presented in Table 2 and Figure 2.

Particle aspect ratios were determined through two-dimensional image analysis in ImageJ v1.54t, based on a minimum of 50 randomly selected particles for each aggregate type. Average aspect ratios were 1.441 for HDPE-G, 1.383 for HDPE-C, and 1.427 for PET-C, indicating similar projected length-to-width ratios across the three plastic aggregate types. However, visual examination revealed clear differences in overall morphology, with HDPE-C and PET-C exhibiting flat, chip-like geometries and HDPE-G exhibiting a more compact, granular morphology. Because the two-dimensional aspect ratio captures only the projected length-to-width relationship, it does not fully resolve the flatness that distinguishes chip-shaped from granular particles. Therefore, a three-dimensional descriptor such as a thickness-to-length flatness index would more directly quantify this distinction and is recommended for future work. Together with the coarser particle size distribution, these morphological differences may influence particle packing, particle–matrix interaction, interfacial defect formation, crack propagation behavior, and transport characteristics within the concrete matrix.

Replacing 10% of the natural fine aggregate by volume with recycled plastic particles in the 2–4 mm size range altered the fine aggregate skeleton because the plastic particles were substantially coarser than the dominant 0.30–0.60 mm natural sand fraction. This difference may affect particle packing, local void distribution, and cement paste distribution around the aggregates. These changes influence the interfacial transition zone (ITZ) characteristics, stress transfer efficiency, crack initiation, and crack propagation and, consequently, may affect the mechanical performance of concrete independently of polymer chemistry. Aggregate gradation has been shown to significantly influence packing density, workability, and the mechanical performance of cementitious composites [28,29], while aggregate size and morphology affect packing efficiency, ITZ development, and stress transfer behavior [30,31]. This gradation mismatch can be quantified directly. Based on the sieve analysis in Table 2, the blended fine aggregate fineness modulus increased from 2.71 for the control mixture to approximately 2.92–2.93 for all recycled plastic mixtures, indicating a similar gradation shift across the HDPE-G, HDPE-C, and PET-C systems. Consequently, comparisons between the recycled plastic mixtures and the control should be interpreted as reflecting the combined effects of aggregate gradation, particle packing, polymer morphology, and interfacial bonding. However, because all recycled plastic mixtures were prepared using the same 2–4 mm particle size range and exhibited nearly identical blended fine aggregate fineness moduli, differences among polymer types and between untreated and treated mixtures cannot be attributed to gradation alone. These differences are more reasonably associated with particle morphology, polymer chemistry, surface treatment, and interfacial bonding. A matched mineral aggregate control prepared using natural mineral aggregate in the same particle size range as the recycled plastic aggregates was not included in the present experimental program. Although the similar blended fineness modulus indicates that gradation remained essentially constant among the recycled plastic mixtures, a size-matched mineral aggregate control would be required to fully decouple the effects of aggregate gradation from polymer-specific interfacial behavior in future studies.

### 2.2. Surface Treatment

The surface condition of PA was evaluated under three states: untreated, oxidative hydrogen peroxide (H_2_O_2_) treatment for HDPE aggregates, and alkaline sodium hydroxide (NaOH) treatment for PET-C. Untreated batches of each plastic type were used as reference conditions to quantify the effectiveness of surface modification.

For HDPE-G and HDPE-C, sieved particles (2–4 mm) were immersed in a 20% H_2_O_2_ solution at 23 ± 2 °C (room temperature) for 24 h. Hydrogen peroxide was selected as the treatment agent because the polyolefin backbone of HDPE is chemically inert toward alkaline hydrolysis, precluding the use of NaOH as an effective surface modifier for this polymer [11]. The 20% treatment concentration was adopted to provide sufficient reactive oxygen species for surface oxidation while remaining within the range reported in cementitious applications [32]. The 24 h treatment duration was determined through preliminary trials to ensure adequate surface modification of the relatively large 2–4 mm particles without observable bulk degradation. After treatment, particles were thoroughly rinsed with distilled water until a neutral pH was achieved and subsequently oven-dried at 60 °C for 24 h. Weight loss after treatment was 0.60% for HDPE-G and 1.80% for HDPE-C. The greater weight loss of HDPE-C may be associated with its flat, thin particle geometry, which provides greater exposed surface area per unit mass than the more compact HDPE-G particles.

For PET-C, sieved particles (2–4 mm) were immersed in a 20 wt.% NaOH solution at room temperature for 96 h. NaOH was selected because the ester linkages in PET undergo alkaline hydrolysis via hydroxide-ion attack, whereas the polyolefin backbone of HDPE remains largely unaffected under similar conditions [11,33]. This concentration falls within the upper range commonly used for alkaline PET surface modification and was chosen to achieve measurable surface activation of relatively large particles under ambient conditions within a practical laboratory treatment period. Because alkaline hydrolysis of PET at room temperature proceeds relatively slowly, the 96 h exposure period was adopted to provide sufficient reaction time while avoiding extensive bulk degradation [33,34]. After treatment, the particles were rinsed with distilled water until a neutral pH was achieved and oven-dried using the same procedure as the HDPE aggregates. The measured weight loss of 3.58% confirms that the treatment produced controlled surface modification without excessive degradation and is consistent with previous studies reporting limited mass loss during alkaline surface treatment [18,19].

The NaOH treatment was designed as an accelerated laboratory surface pretreatment rather than a simulation of the chemical environment of hydrated Portland cement. Conventional alkaline hydrolysis of PET employs NaOH or KOH solutions over a broad concentration range, with treatment conditions selected to control the extent of hydrolysis and surface activation rather than reproducing service conditions [33]. Under alkaline conditions, hydroxide ions cleave PET ester linkages, generating oxygen-containing surface functional groups and increasing surface roughness, both of which may enhance physicochemical interaction and mechanical interlocking with the cement paste. Because the present study employed relatively large (2–4 mm) PET particles treated at room temperature, a comparatively high hydroxide concentration and a 96 h exposure period were required to achieve measurable surface modification within a practical laboratory timescale. The measured 3.58% weight loss further indicates that the treatment primarily modified the particle surface rather than causing substantial bulk degradation. The alkaline environment of hydrated cement differs fundamentally from that of the laboratory pretreatment. The pore solution of Portland cement is highly alkaline due to dissolved Na^+^, K^+^, and Ca (OH)_2_; however, its hydroxide concentration is considerably lower than that of the 20 wt.% NaOH treatment solution [16,35]. Accordingly, a saturated Ca (OH)_2_ solution or simulated cement pore solution was not used because these media are primarily intended to evaluate the long-term alkaline durability of polymeric materials under representative service conditions rather than produce controlled surface modification before concrete production. In contrast, the concentrated NaOH solution used in this study was intended to establish the desired surface characteristics before mixing. Although continued alkaline hydrolysis of PET after incorporation into concrete cannot be completely ruled out, previous studies have shown that degradation in simulated cement pore solution occurs gradually over extended exposure periods and is substantially slower than under concentrated NaOH treatment [16,35]. The long-term stability of the treated PET–cement interface therefore remains an important topic for future investigation.

The effectiveness of the surface treatments was subsequently evaluated through FTIR characterization of the untreated and treated plastic particles and SEM–EDS examination of the plastic–cement paste interfaces.

Although the proposed surface treatments improve the engineering performance of recycled plastic aggregate concrete, they also introduce additional environmental and economic burdens due to chemical production, extended treatment time, rinsing water, oven drying, and wastewater management. To provide a preliminary assessment of these trade-offs, Table 3 presents a simplified screening inventory of estimated treatment chemical consumption and the production-stage carbon footprint per cubic meter of concrete, using the treatment bath liquid-to-solid ratio (1.5:1) adopted in this study. Hydrogen peroxide is predominantly manufactured via the anthraquinone oxidation process, whereas sodium hydroxide is produced through the chlor-alkali electrolysis process. Both are energy-intensive processes that contribute additional production-stage greenhouse gas emissions [36,37]. Accordingly, hydrogen peroxide and sodium hydroxide production generate approximately 3.92 and 0.63 kg CO_2-eq_/kg, respectively [36,37]. Based on the estimated chemical consumption, the treatment-related carbon footprint exceeded the carbon footprint avoided through natural sand replacement, particularly for the H_2_O_2_-treated mixtures. Therefore, the proposed surface treatments should not be considered environmentally beneficial from a production-stage carbon perspective alone. This finding highlights the importance of evaluating sustainability using a holistic life-cycle perspective rather than relying solely on production-stage greenhouse gas emissions [38]. Instead, their potential sustainability benefit lies in improving the engineering performance of recycled plastic aggregate concrete, thereby facilitating greater utilization of recycled plastic waste and potentially extending concrete service life. From a practical perspective, the treatment process also introduces additional costs associated with chemical procurement, extended treatment time, water consumption, oven drying, wastewater treatment, and labor. Because the present study focused on technical feasibility, these environmental and economic aspects were evaluated qualitatively. A comprehensive life cycle and techno-economic assessment is therefore recommended for future work to determine whether the long-term engineering and environmental benefits justify the additional processing requirements of the proposed treatment process.

### 2.3. Concrete Mix Design

A single baseline concrete mix proportioned on an absolute volume basis of a 0.5 water-to-cement (w/c) ratio was used as a reference for all experimental groups. Plastic aggregate replaced natural sand at 10% by volume, yielding seven mix groups in total. This replacement level was selected because it is expected to produce a measurable but recoverable performance penalty with recycled plastic aggregate, allowing the effectiveness of surface treatment to be evaluated clearly. Frigione [12] reported an approximately 30% reduction in compressive strength at a comparable 10% PET fine-aggregate replacement level without surface treatment. Higher replacement levels have been shown to produce disproportionately larger strength losses. Ten percent was therefore chosen as a level severe enough to reveal the mechanistic effects of particle morphology and interfacial bonding, while remaining within a range where surface treatment could plausibly restore performance to acceptable limits. Plastic type and treatment conditions are summarized in Table 4; absolute mix proportions are presented in Table 5. No chemical admixtures were used in this study. After casting, all specimens were demolded after 24 h and subsequently cured in water maintained at 23 ± 2 °C until the specified testing age.

## 3. Test Methods

### 3.1. Slump

The slump of each concrete mixture was measured immediately after mixing in accordance with ASTM C143 [39]. Fresh concrete was placed into a standard slump cone in three equal layers, with each layer consolidated using 25 strokes of a tamping rod. After the cone was carefully lifted vertically, the slump was determined by measuring the reduction in the concrete’s height.

### 3.2. Fresh Concrete Density

Fresh concrete density was measured in accordance with ASTM C138 [40]. A rigid, watertight cylindrical measure of known, calibrated volume was used, meeting the minimum capacity requirement specified in ASTM C29 [41] for the nominal maximum aggregate size. The empty measure was weighed and then filled with fresh concrete in three approximately equal layers. Each layer was consolidated by rodding with 25 strokes of a tamping rod. The filled measure was then weighed, and the density was calculated by dividing the net mass of the concrete, the difference between the filled and empty measure weights, by the calibrated volume of the measure. Three replicate measurements were conducted for each mixture, and the results are reported as the mean with the corresponding standard deviation.

### 3.3. Mechanical Properties

#### 3.3.1. Compression Strength

Compressive strength was evaluated in accordance with ASTM C39 [42] using cylindrical specimens measuring 102 mm × 204 mm (4 in. × 8 in.). Specimens were tested after 3, 7, and 28 days of curing. For each mixture at each testing age, three cylinders were tested, and the compressive strength was reported as the average of the three measurements. All specimens were continuously loaded at 0.22 MPa/s (31.8 psi/s) until failure, and the average peak compressive strength was recorded.

#### 3.3.2. Splitting Tensile Strength

Splitting tensile strength was measured at 28 days using a material testing system (MTS). Cylindrical specimens were loaded at a constant displacement rate of 1 mm/min until failure, and the peak load was recorded. The splitting tensile strength was calculated using Equation (1):

(1) $$f_{t}=\frac{2P}{\pi LD}$$

where $f_{t}$ is the splitting tensile strength (MPa), P is the peak load at failure (N), L is the specimen length (mm), and D is the specimen diameter (mm). Three specimens were tested for each concrete mixture, and the reported value represents the average splitting tensile strength.

#### 3.3.3. Flexural Strength

Flexural strength was evaluated at 28 days in accordance with ASTM C78 [43] using beam specimens measuring 102 mm × 102 mm × 381 mm (4 in. × 4 in. × 15 in.). The specimens were tested under third-point loading on an MTS. Loading was applied continuously at the rate specified in ASTM C78 [43] until failure. Three beam specimens were tested for each concrete mixture, and the reported flexural strength is the average of the three test results. The flexural strength test was performed for only 28 days because the objective of this study was to compare the mature flexural performance of concrete incorporating untreated and surface-treated plastic fine aggregates. Since flexural strength is commonly specified and evaluated at the standard 28-day curing age for structural concrete, early-age flexural strength measurements were not included in the scope of this investigation. Future studies may further evaluate the evolution of early-age flexural strength to characterize the influence of plastic aggregate surface treatment.

#### 3.3.4. Modulus of Elasticity

The static chord modulus of elasticity was measured at 28 days in accordance with ASTM C469 [44] using three cylindrical specimens from each concrete mixture. Axial deformation was monitored using a compressometer fitted with a longitudinal displacement gauge mounted at the mid-height of each specimen. Compression testing was performed at the same loading rate as the compressive strength test. The static chord modulus of elasticity was calculated using Equation (2), which defines the slope of the stress–strain curve within the elastic region:

(2) $$E_{c}=\frac{S_{2}-S_{1}}{\varepsilon_{2}-0.000050}$$

where $E_{c}$ is the static chord modulus of elasticity (MPa), $S_{2}$ is the stress corresponding to 40% of the ultimate compressive load, $S_{1}$ is the stress associated with a longitudinal strain of 50 με (0.000050), and $\varepsilon_{2}$ is the longitudinal strain measured at $S_{2}$. The reported modulus of elasticity for each mixture represents the average of three specimens.

#### 3.3.5. Ultrasonic Pulse Velocity (UPV)

Ultrasonic pulse velocity (UPV) was measured at 28 days in accordance with ASTM C597 [45] using the direct transmission method. Test specimens measuring 102 mm in diameter and 30 mm in thickness were obtained by sectioning cylindrical concrete specimens. A PicoScope data acquisition system (Pico Technology Limited, St Neots, UK) equipped with two 50 kHz piezoelectric transducers was used for the measurements. The transducers were positioned on opposite faces of each specimen with an ultrasonic coupling gel applied to ensure effective acoustic contact. The pulse transit time was recorded, and the ultrasonic pulse velocity was calculated from the measured travel time and specimen thickness. Three specimens were tested for each concrete mixture, and the reported UPV value represents the average of the three measurements.

### 3.4. FTIR and SEM–EDS Characterization

Fourier-transform infrared spectroscopy was performed on untreated and treated HDPE-G and PET-C particles using an IRTracer-100 FTIR spectrometer (Shimadzu Corporation, Kyoto, Japan) over a wavenumber range of 400–4000 cm^−1^ to evaluate treatment-induced changes in surface functional groups. The plastic–cement paste interfaces of untreated and treated plastic concrete specimens were examined using a Thermo Fisher Scientific Apreo 2 field-emission scanning electron microscope (Hillsboro, OR, USA) operated at an accelerating voltage of 20 kV. SEM imaging was used to examine interfacial morphology and plastic–paste contact, while EDS analysis was used to evaluate local elemental composition at the interfacial region.

### 3.5. Rapid Chloride Penetrability Test

Chloride ion penetrability was assessed in accordance with ASTM C1202 [46] at 28 days of curing. Disc specimens (Ø102 × 51 mm) were cut from the mid-section of cylindrical samples, vacuum-saturated for 18 h, and subjected to a 60 V direct-current potential for 6 h. The total charge passed (in coulombs) was recorded as the measure of chloride ion penetrability. Three replicate specimens were tested per mixture at each age, and the reported value represents the average of the three measurements.

### 3.6. Surface Electrical Resistivity

Surface electrical resistivity was measured in accordance with AASHTO T358 [47] using the Wenner four-electrode method at curing ages of 3, 7, and 28 days. Three cylindrical specimens from each concrete mixture were tested at each age. For each specimen, eight longitudinal measurements were obtained at 45° intervals around the circumference to minimize the influence of directional variability, and the average value was used for analysis. Measurements were performed using a resistivity meter with a probe spacing of 38 mm. The electrical resistivity was calculated from the measured resistance while accounting for the specimen geometry and probe configuration.

### 3.7. Water Absorption and Permeable Voids

Water absorption and the volume of permeable voids were measured in accordance with ASTM C642 [48] using disc specimens prepared from 102 mm diameter concrete cylinders after 28 days of curing. The specimens were first oven-dried at 110 ± 5 °C to a constant mass, cooled to room temperature, and weighed to obtain the oven-dry mass. They were subsequently immersed in water for 48 h to determine the saturated-surface-dry (SSD) mass. The specimens were then boiled in water for 5 h, allowed to cool to room temperature while remaining immersed, and weighed to obtain the boiled saturated mass. These mass measurements were used to calculate water absorption and the volume of permeable voids in accordance with ASTM C642.

### 3.8. Freeze–Thaw Durability

Freeze–thaw resistance was evaluated in accordance with ASTM C666 Procedure A [49] using beam specimens measuring 76 mm × 102 mm × 381 mm (3 in. × 4 in. × 15 in.). Following 14 days of water curing, the specimens were exposed to repeated freezing and thawing cycles in a Humboldt freeze–thaw chamber (Humboldt Mfg. Co., Elgin, IL, USA), with temperatures ranging from approximately −18 °C to +4 °C. The fundamental transverse frequency and specimen length were measured every 30 cycles to monitor changes in dynamic stiffness and dimensional stability through the standard 300-cycle terminal count specified by ASTM C666. The relative dynamic modulus of elasticity (RDM), length change, and durability factor (DF) were calculated in accordance with ASTM C666, using M = 300 as the specified terminal cycle count. No specimen fell below the 60% RDM failure criterion at any point during testing, so N = M = 300 for all mixtures.

### 3.9. Drying Shrinkage

Drying shrinkage was measured in accordance with ASTM C157 [50] using concrete prism specimens measuring 75 mm × 75 mm × 285 mm (3 in. × 3 in. × 11.25 in.) and a length comparator conforming to ASTM C490. After 28 days of moist curing, the specimens were transferred to a controlled environment maintained at 23 ± 2 °C and 50 ± 4% relative humidity to initiate drying. Length measurements were obtained periodically from day 1 through day 28 of drying. Three specimens were tested for each concrete mixture, and the reported drying shrinkage values represent the average of the three measurements. The mixture proportions were identical to those listed in Table 5 to ensure consistency with the other concrete performance tests conducted in this study.

### 3.10. Microscopic Analysis

Optical microscopy was performed to examine fracture characteristics and the plastic–cement interfacial transition zone (ITZ) using cross-sections obtained from beam specimens after flexural testing. Sections were cut from the mid-span failure region and then ground and polished to expose the internal microstructure. Images of the control, HDPE-G, HDPE-C, and PET-C mixtures were captured at a constant magnification with a 1 mm scale bar. The micrographs were analyzed to evaluate crack propagation patterns, fracture morphology, and the extent of interfacial debonding between the plastic particles and the surrounding cement matrix.

### 3.11. Statistical Analysis

Statistical comparisons between untreated and treated recycled plastic aggregate mixtures were performed using two-tailed Welch’s *t*-tests based on three replicate specimens. Statistical significance was evaluated at a significance level of *p* < 0.05. Differences with *p* < 0.05 were considered statistically significant, while differences with *p* ≥ 0.05 were interpreted as numerical trends without statistical confirmation.

## 4. Test Results and Discussion

### 4.1. Slump

**Figure 3:**
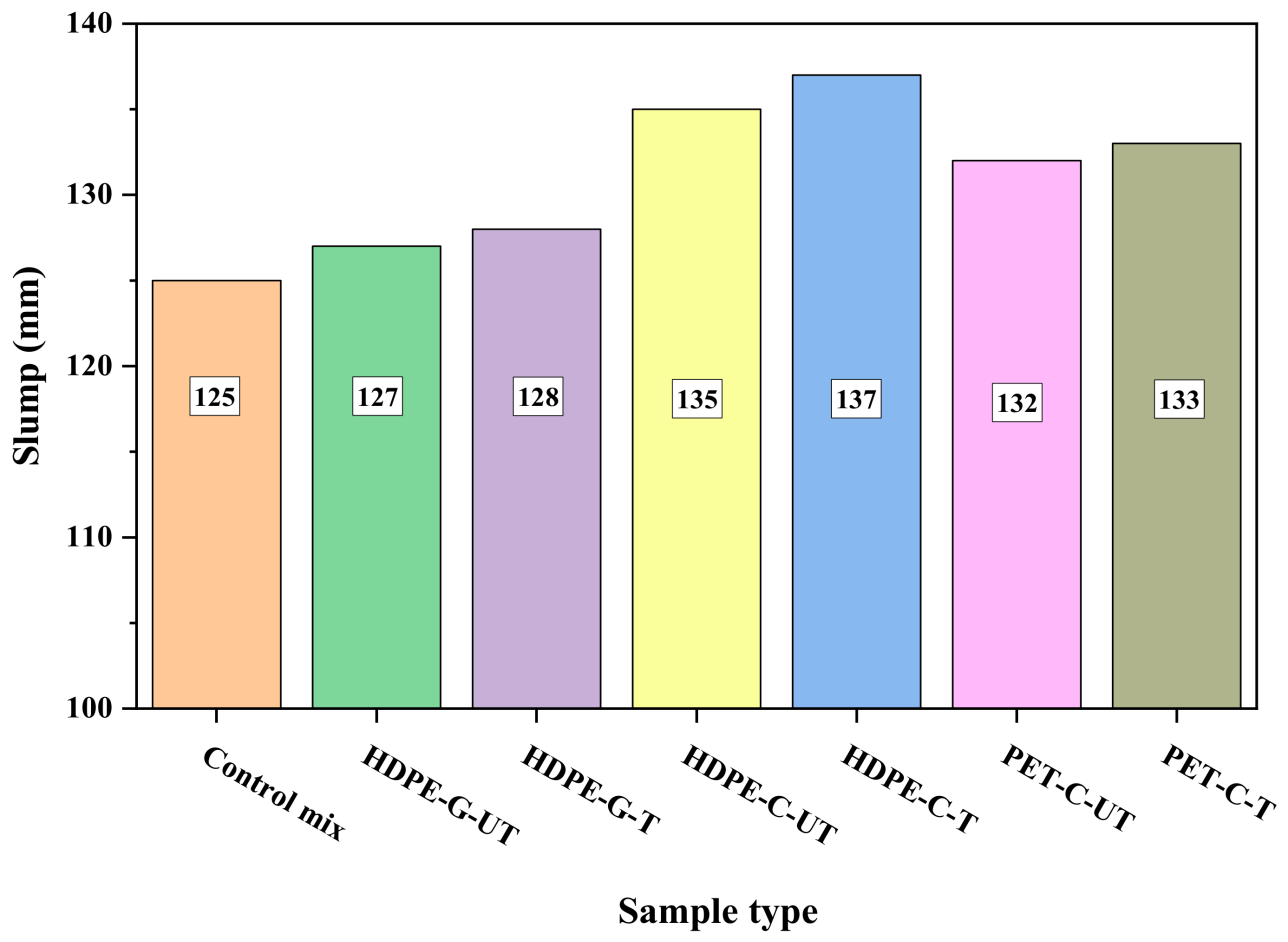
Slump of control and recycled plastic aggregate concrete mixtures with and without surface treatment.

Figure 3 shows that the slump ranged from 125 mm for the control mixture to 137 mm for the HDPE-C-T mixture, with all recycled plastic aggregate concretes exhibiting higher workability than the control. This consistent increase is attributed to the combined effects of the hydrophobic and essentially non-absorptive nature of the recycled plastic particles and the coarser blended fine aggregate gradation. The negligible water absorption of the plastic particles retains a greater proportion of the mixing water as free water, while the coarser blended gradation reduces the specific surface area requiring paste coverage, together increasing the lubricating water–paste film and improving workability. Unlike natural sand, recycled plastic aggregates exhibit negligible water absorption, thereby reducing paste demand and increasing the thickness of the lubricating water–paste film surrounding the particles, which lowers internal friction during flow. In addition, replacing the dominant natural sand fraction (0.3–0.6 mm) with coarser recycled plastic particles (2–4 mm) reduced the specific surface area of the blended fine aggregate skeleton, decreasing the amount of paste required for particle coating and further enhancing workability. This interpretation is consistent with the aggregate packing concepts reported by Cook and Ley [51] and previous studies on recycled plastic aggregates [11]. Particle morphology also influenced slump. The chip-shaped HDPE-C mixtures consistently produced slightly higher slump than the granular HDPE-G mixtures, likely because their flatter geometry reduced interparticle friction and facilitated particle movement during flow. PET-C mixtures exhibited intermediate slump values, suggesting that the beneficial effect of chip morphology was partially offset by the higher density of PET particles. Surface treatment produced only marginal changes in slump of approximately 1–2 mm, indicating that fresh concrete behavior was governed primarily by particle geometry, gradation, and water demand rather than the applied surface treatment.

### 4.2. Fresh Concrete Density

**Figure 4:**
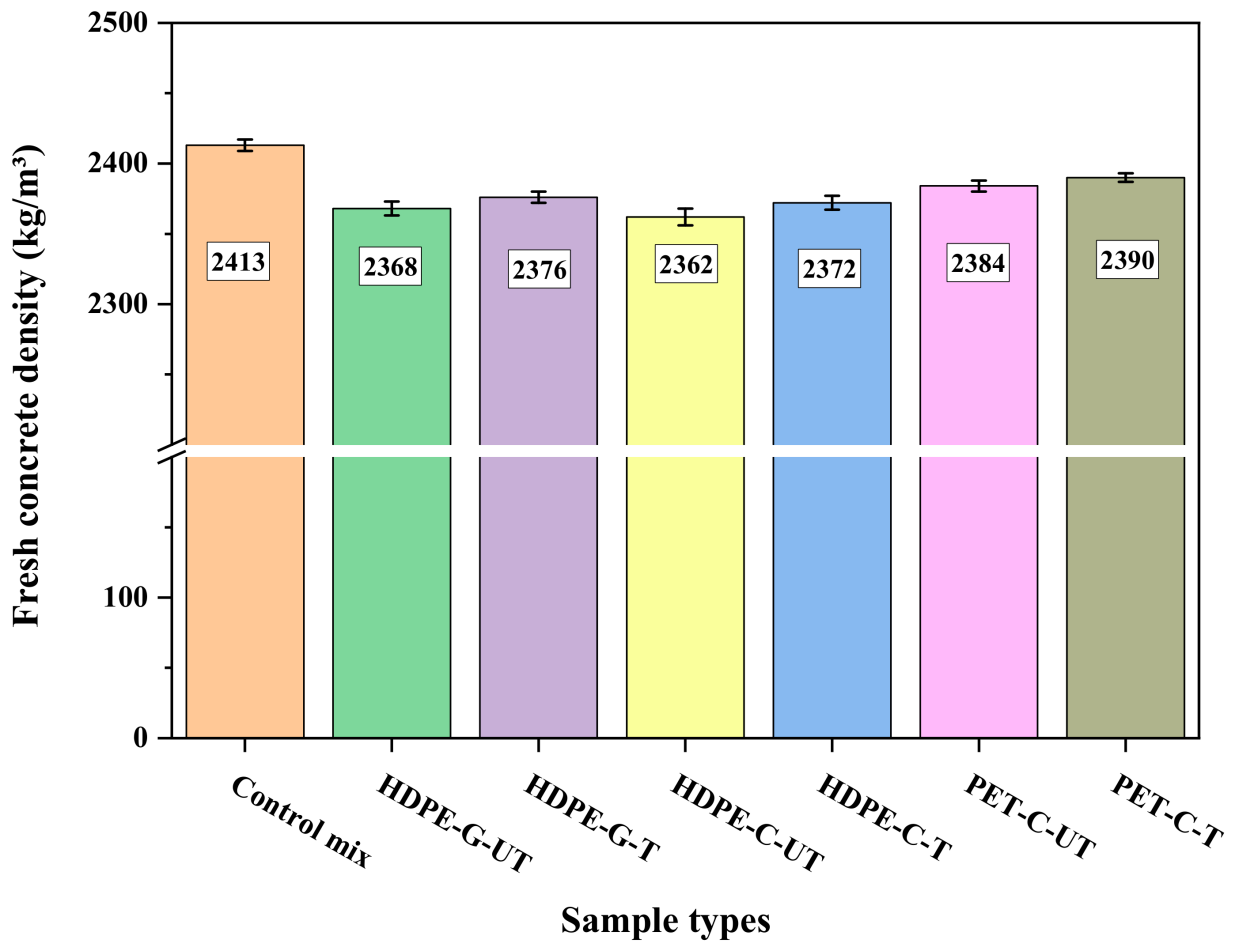
Fresh concrete density of control and recycled plastic aggregate concrete mixtures with and without surface treatment.

Figure 4 presents the fresh concrete densities of all mixtures. The recycled plastic aggregate concrete exhibited densities ranging from 2362 to 2390 kg/m^3^, compared with 2413 kg/m^3^ for the control mixture. This reduction is governed primarily by the rule of mixtures, in which the composite density reflects the densities and volumetric proportions of its constituent materials. Replacing 10% of the natural fine aggregate with recycled HDPE or PET therefore reduced the overall concrete density because both polymers have substantially lower specific gravity than natural sand (Table 1). The decrease remained relatively small (approximately 1–2%) because only a limited fraction of the fine aggregate was replaced, while most of the concrete volume continued to consist of coarse aggregate, cement paste, and natural mineral aggregates, which dominate the composite density. Polymer type also influences fresh density. The HDPE mixtures consistently exhibited lower densities than the PET mixtures because HDPE has a considerably lower specific gravity (0.95) than PET (1.39). Surface treatment produced negligible changes within each polymer type, indicating that H_2_O_2_ and NaOH treatments modified the surface chemistry without significantly altering the bulk density or volume of the recycled plastic particles. Similar modest reductions in fresh concrete density following partial replacement of natural fine aggregate with recycled plastic have been reported in previous studies, confirming that density changes are governed primarily by polymer density and replacement level rather than surface treatment.

### 4.3. Mechanical Properties

#### 4.3.1. Compression Strength

**Figure 5:**
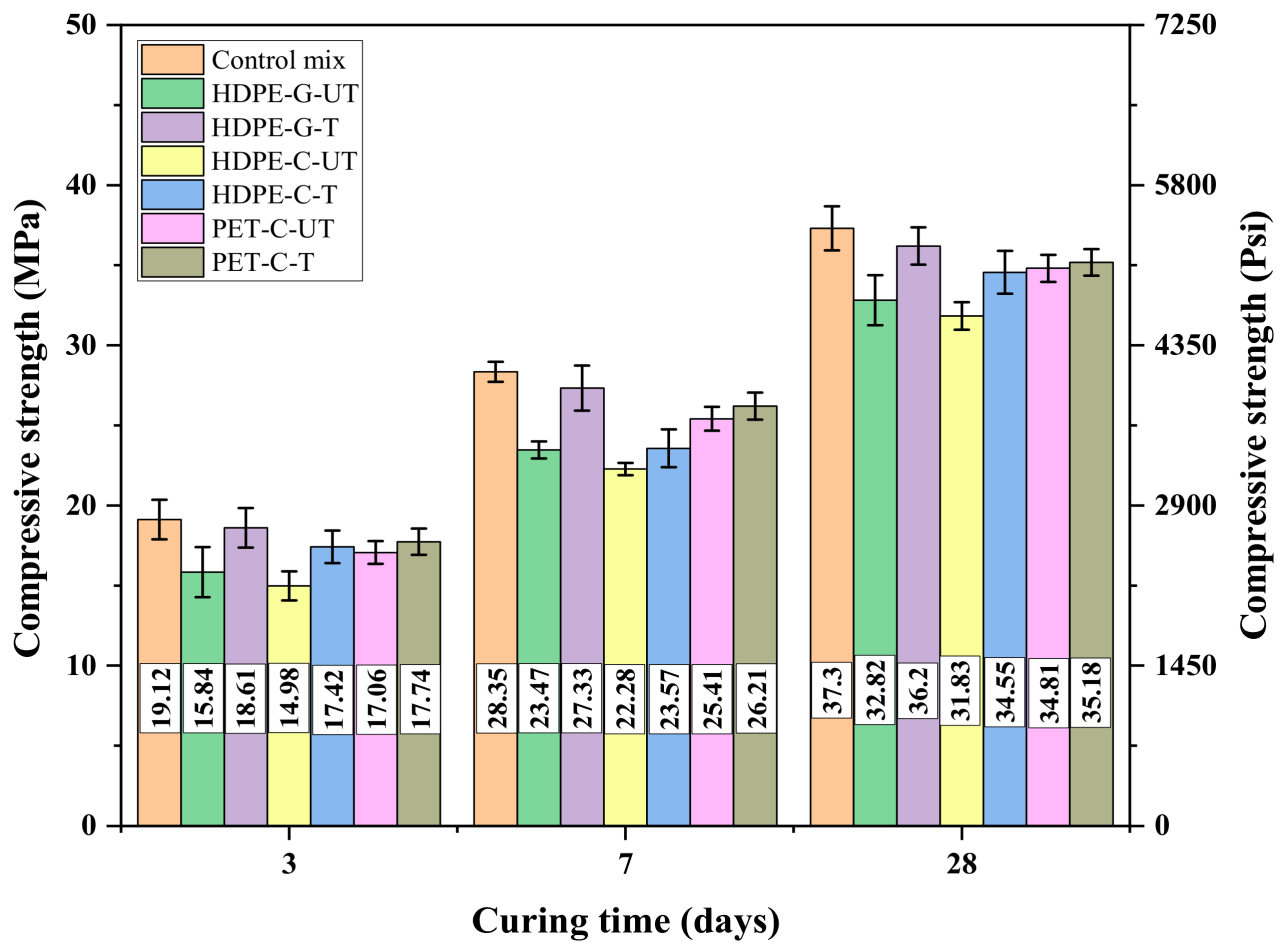
Compressive strength of control and recycled plastic aggregate concrete mixtures at 3, 7, and 28 days of curing.

Figure 5 shows the development of compressive strength in the control and recycled plastic aggregate concretes at 3, 7, and 28 days. All mixtures exhibited continuous strength gain with curing age as cement hydration progressed and the cement matrix densified. However, replacing 10% of the natural fine aggregate with untreated recycled plastic reduced compressive strength at all curing ages. At 28 days, the untreated mixtures achieved strengths ranging from 31.83 to 34.81 MPa, compared with 37.30 MPa for the control. This reduction is attributed to the combined influence of the coarser recycled plastic aggregate gradation and the weaker plastic–cement ITZ. Relative to the control, the gradation mismatch may alter particle packing and stress-transfer efficiency, while the lower stiffness and weaker interfacial bonding of the recycled plastic particles further promote stress concentration and microcrack initiation within the surrounding ITZ. Because recycled plastic particles have lower elastic stiffness than natural mineral aggregates, compressive stresses are transferred less efficiently across the interface, promoting stress concentration and microcrack initiation within the surrounding ITZ rather than through the aggregate itself. Similar behavior has been reported for recycled plastic aggregate concrete, where interfacial bonding governs compressive strength more strongly than the intrinsic strength of the polymer. Particle morphology further influenced the compressive response. The chip-shaped HDPE-C-UT mixture exhibited the lowest compressive strength, whereas HDPE-G-UT retained comparatively higher strength. This trend aligns with the higher permeable void content measured for HDPE-C-UT, indicating a more porous and less continuous ITZ. Surface treatment produced numerical increases in the mean compressive strength of all recycled plastic mixtures. At 28 days, H_2_O_2_ treatment increased the mean compressive strength of HDPE-G and HDPE-C by approximately 10.3% and 8.5%, respectively, while NaOH treatment increased PET-C by only 1.1%. However, the differences between the untreated and treated mixtures were not statistically significant, with *p* = 0.057 for HDPE-G, *p* = 0.059 for HDPE-C, and *p* = 0.625 for PET-C. Therefore, these increases are interpreted as numerical trends rather than statistically confirmed improvements in compressive strength.

#### 4.3.2. Splitting Tensile Strength

**Figure 6:**
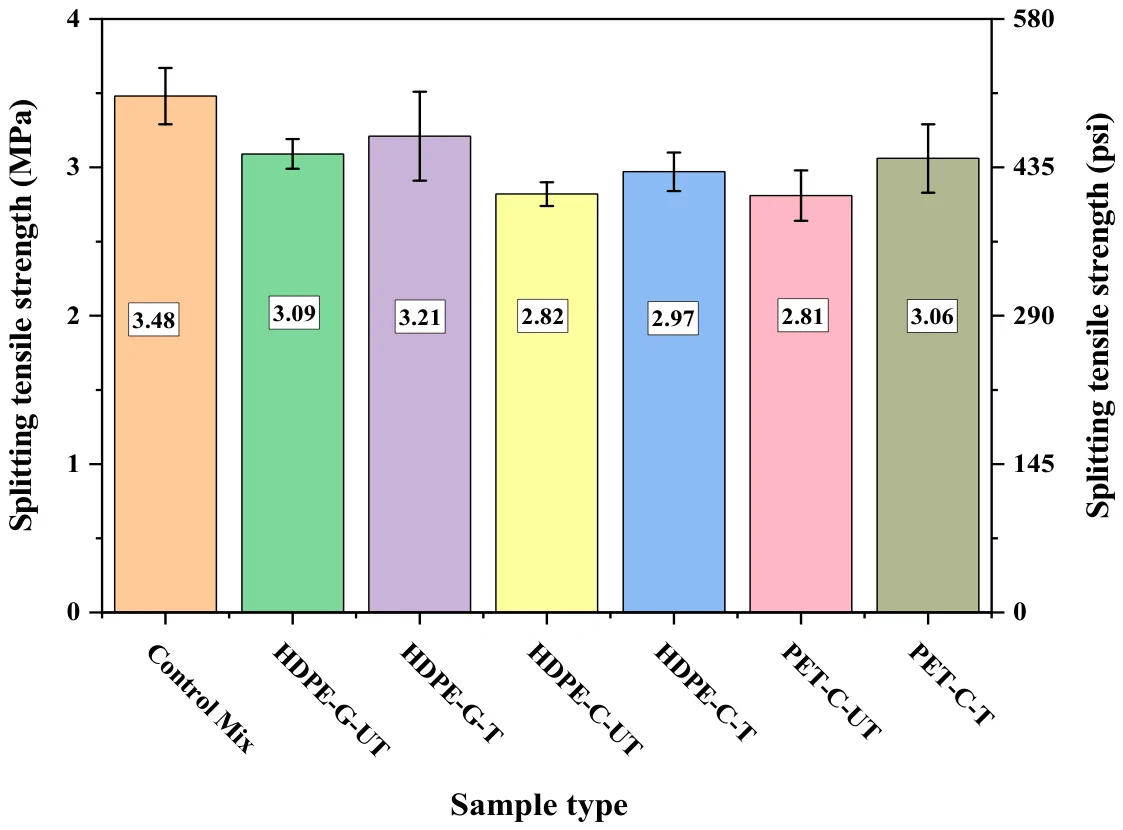
Splitting tensile strength of control and recycled plastic aggregate concrete mixtures at 28 days of curing.

**Figure 7:**
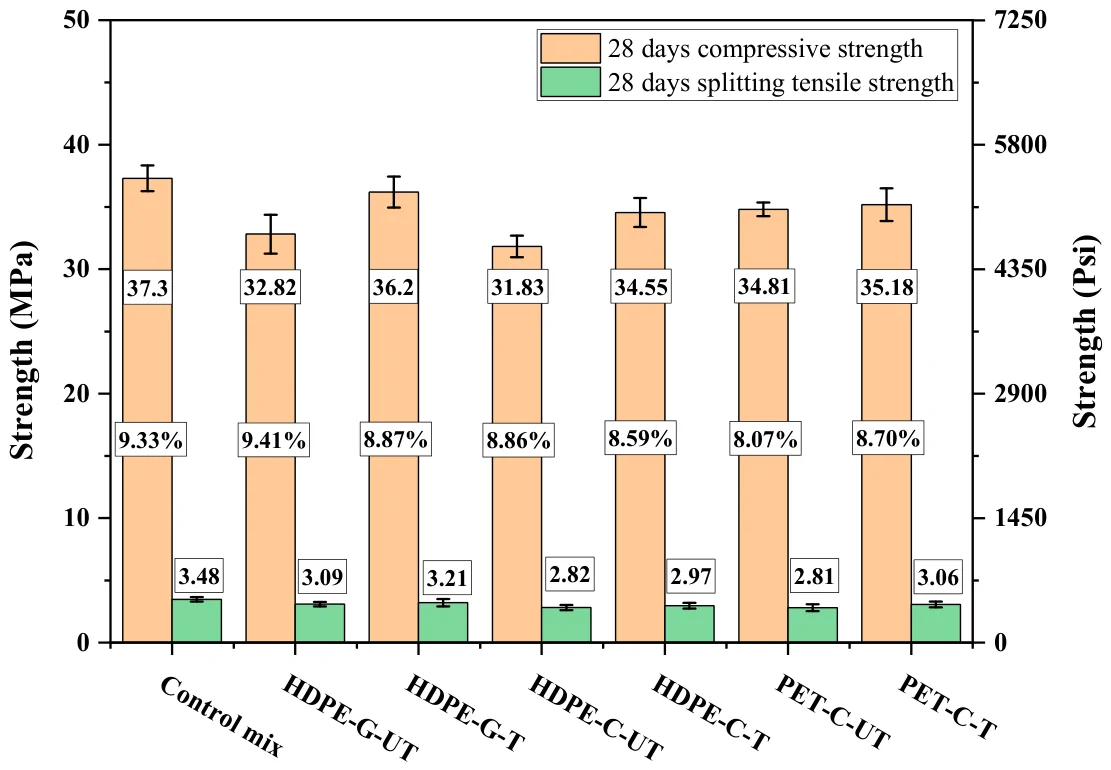
Ratios of 28-day compressive and splitting tensile strengths.

Figure 6 presents the 28-day splitting tensile strength of the control and recycled plastic aggregate concrete. Incorporating untreated recycled plastic aggregates reduced splitting tensile strength relative to the control, with HDPE-C-UT and PET-C-UT exhibiting the lowest values. This reduction is primarily attributed to the lower stiffness of the recycled plastic particles and the weaker plastic–cement ITZ, which promotes crack initiation and propagation under indirect tensile loading. Unlike compressive loading, splitting tensile failure is governed primarily by fracture through the weakest interfacial regions; consequently, tensile strength is particularly sensitive to ITZ quality and particle–paste bonding. HDPE-G-UT retained higher tensile strength than HDPE-C-UT, indicating that particle morphology also influenced crack resistance. This observation is consistent with the higher permeable void content measured for the HDPE-C mixtures, suggesting a more porous and discontinuous ITZ. Surface treatment produced numerical increases in the mean splitting tensile strength of all recycled plastic mixtures. Relative to their untreated counterparts, the mean splitting tensile strength increased by approximately 3.9% for HDPE-G, 5.3% for HDPE-C, and 8.9% for PET-C. However, these differences were not statistically significant, with *p* = 0.057 for HDPE-G, *p* = 0.187 for HDPE-C, and *p* = 0.227 for PET-C. Therefore, the observed increases are interpreted as numerical trends rather than statistically confirmed improvements in splitting tensile strength. The higher mean strengths of the treated mixtures are consistent with the improved interfacial interaction observed in the SEM analysis, although the statistical results do not establish a significant treatment effect on splitting tensile strength. As shown in Figure 7, the corresponding ft/fc ratios remained within 8.07–9.41%, which falls within the typical range of 8–12% reported for conventional concrete [52], indicating that recycled plastic aggregates altered the magnitude of tensile strength without fundamentally changing its relationship with compressive strength.

#### 4.3.3. Flexural Strength

**Figure 8:**
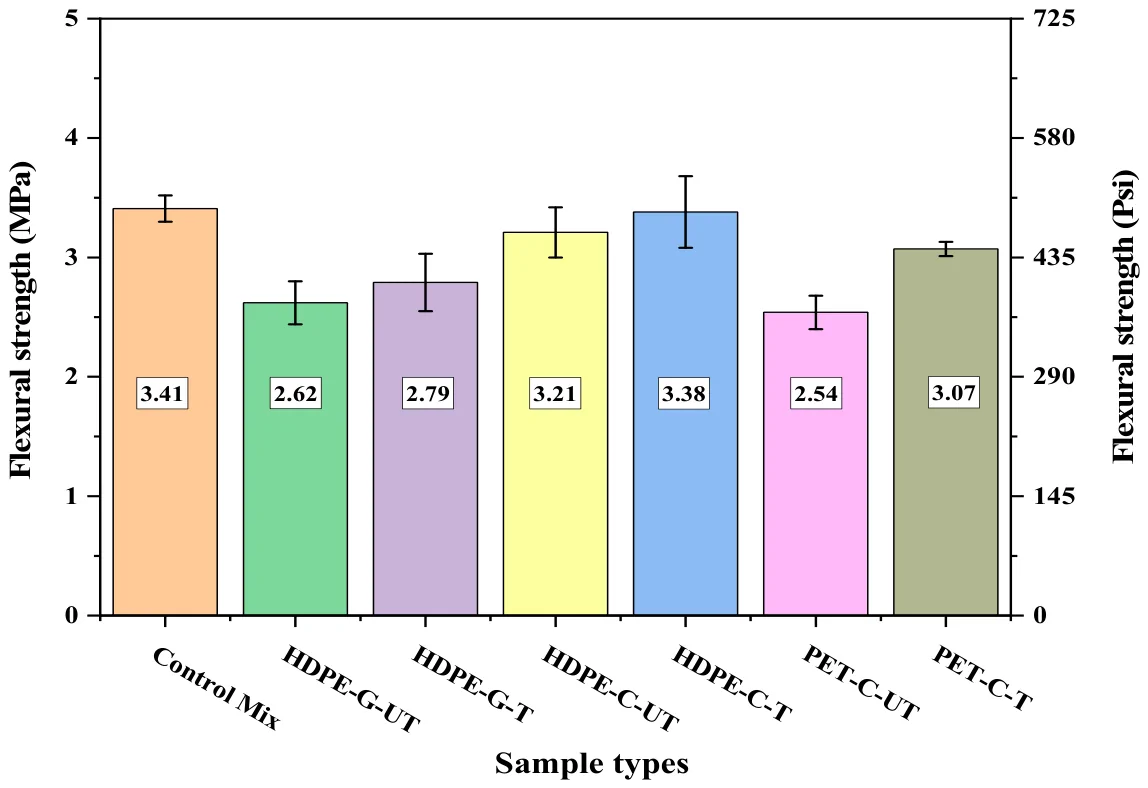
Flexural strength of control and recycled plastic aggregate concrete mixtures at 28 days of curing.

**Figure 9:**
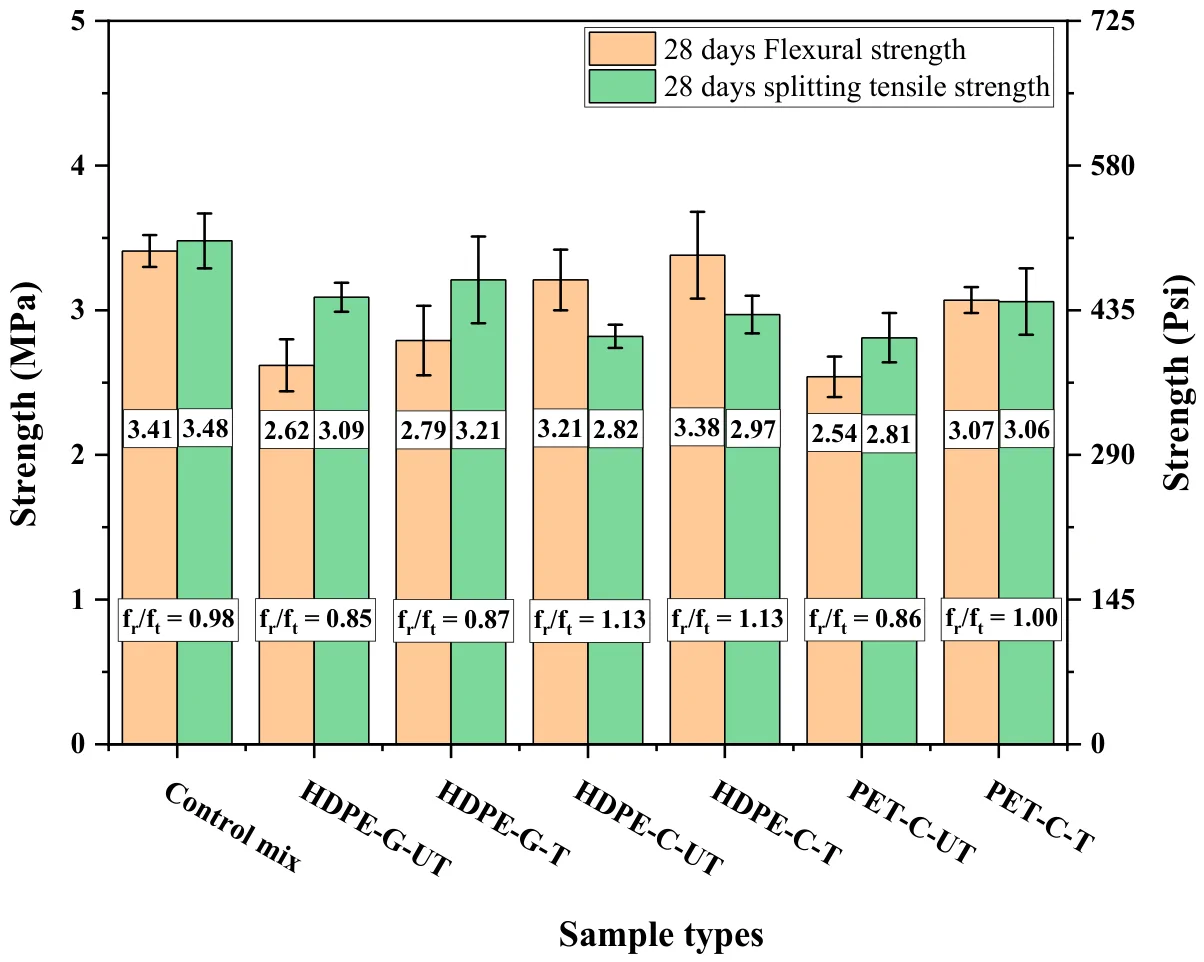
Ratios of 28-day flexural strength and splitting tensile strengths.

Figure 8 shows the 28-day flexural strength. Among the untreated mixtures, HDPE-G-UT exhibited the lowest flexural strength, whereas HDPE-C-UT maintained a substantially higher value. This is the reverse of the ranking observed for compressive strength, where HDPE-G-UT exceeded HDPE-C-UT. This reversal is consistent with particle geometry influencing flexural and compressive behavior through different mechanisms. Under compression, the larger interfacial contact area of the flat HDPE-C particles promotes debonding and strength loss, whereas under flexure, the same flat geometry oriented across the beam’s tension zone instead bridges the crack path, partially offsetting the weaker bond. Surface treatment produced numerical increases in the mean flexural strength of all recycled plastic mixtures, although the statistical significance depended on polymer type. H_2_O_2_ treatment increased the mean flexural strength of HDPE-G and HDPE-C by 6.5% and 5.3%, respectively. However, these differences were not statistically significant, with *p* = 0.398 for HDPE-G and *p* = 0.480 for HDPE-C. In contrast, NaOH treatment increased the mean flexural strength of PET-C by 20.9%, and this increase was statistically significant with *p* = 0.026. These results indicate that the treatment-related improvement in flexural strength was statistically supported by PET-C, while the increases observed for the HDPE mixtures represent numerical trends without statistical confirmation. Among the treated mixtures, HDPE-C-T achieved the highest flexural strength, approaching that of the control. Figure 9 compares the 28-day flexural and splitting tensile strengths of the investigated concrete mixtures. The flexural-to-splitting tensile strength ratio (fr/ft) ranged from 0.85 to 1.13, with untreated HDPE-G and PET-C below unity throughout and HDPE-C mixtures above unity in both treatment states, consistent with the geometry-dependent flexural response described above. Surface treatment increased both flexural and splitting tensile strengths with only minor changes in the fr/ft ratio, suggesting that improved interfacial bonding enhanced crack resistance under both loading conditions without fundamentally altering the underlying tensile behavior of the concrete.

#### 4.3.4. Modulus of Elasticity

**Figure 10:**
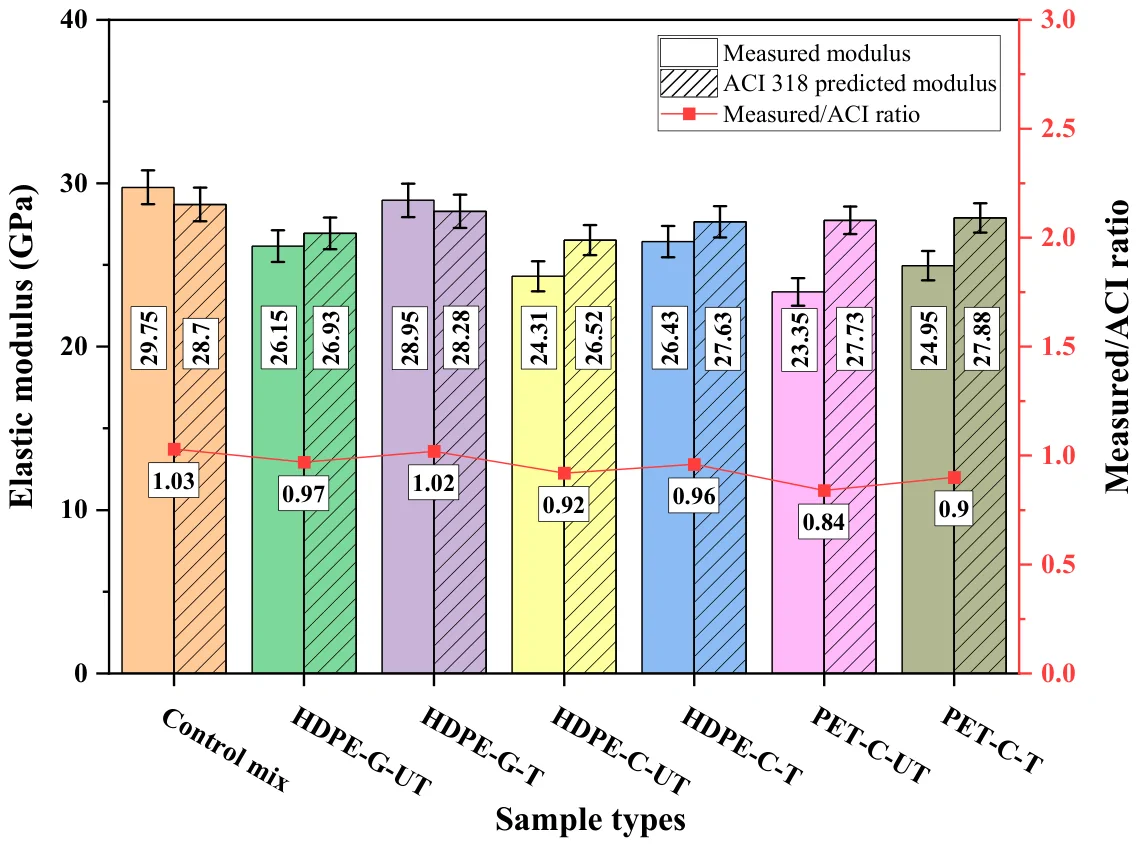
The 28-day static elastic modulus of control and recycled plastic aggregate concrete mixtures, compared with that predicted by ACI 318.

Figure 10 presents the measured 28-day static elastic modulus together with the values predicted by ACI 318. Incorporating untreated recycled plastic aggregates reduced the elastic modulus through the combined influence of the coarser recycled plastic aggregate gradation, the lower intrinsic stiffness of the plastic particles, and less efficient stress transfer across the plastic–cement ITZ. Relative to the control, the gradation mismatch may reduce packing efficiency, whereas improved interfacial bonding following surface treatment partially restored stiffness by enhancing stress transfer across the interface. Particle morphology also influenced the elastic response, with HDPE granules retaining higher stiffness than chip-shaped particles. Surface treatment produced numerical increases in the mean elastic modulus of all recycled plastic mixtures, although the statistical significance depended on polymer type. H_2_O_2_ treatment increased the mean elastic modulus of HDPE-G and HDPE-C by approximately 10.7% and 8.7%, respectively, while NaOH treatment increased PET-C by approximately 6.9%. Statistical analysis showed that the increase for HDPE-G was significant, with *p* = 0.041, whereas the differences for HDPE-C, with *p* = 0.070, and PET-C, with *p* = 0.109, were not statistically significant. Therefore, the treatment-related improvement in elastic modulus was statistically supported for HDPE-G, while the increases observed for HDPE-C and PET-C represent numerical trends without statistical confirmation. The recovery was most complete for H_2_O_2_-treated HDPE-G, which reached within 3% of the control, the closest match to control stiffness among all treated mixtures. The modulus of elasticity generally followed the trend of compressive strength. A similar trend was observed in the ultrasonic pulse velocity results, with mixtures exhibiting higher elastic modulus generally showing higher pulse velocities.

Comparing measured modulus with ACI 318 predictions reveals a polymer-dependent pattern rather than uniform agreement. For the control and both HDPE aggregate types, the measured/ACI ratio remained close to unity, indicating that the empirical strength-based correlation remains broadly applicable. For PET-C, however, the ratio was consistently lower, with the measured modulus falling 10–16% below the ACI 318 prediction in both treatment states. This systematic deviation indicates that PET’s intrinsic stiffness is not fully captured by a correlation calibrated for normal-weight mineral aggregate. Surface treatment narrowed the gap but did not close it. The reduction in elastic modulus with untreated PA incorporation aligns with classical two-phase composite micromechanics. Because the plastic phase is far more compliant than the cement paste and natural aggregate it displaces, composite-bound models such as the Voigt and Reuss limits and intermediate formulations such as the Eshelby and Mori-Tanaka methods predict a reduction in effective composite modulus that scales with the inclusion phase’s volume fraction and stiffness contrast, independent of interfacial bonding quality [53]. Surface treatment does not directly alter this volume fraction-driven stiffness reduction. Instead, it recovers modulus by improving stress transfer efficiency across the plastic paste interface, consistent with the improved bonding reflected in the crack propagation patterns.

#### 4.3.5. Ultrasonic Pulse Velocity (UPV)

**Figure 11:**
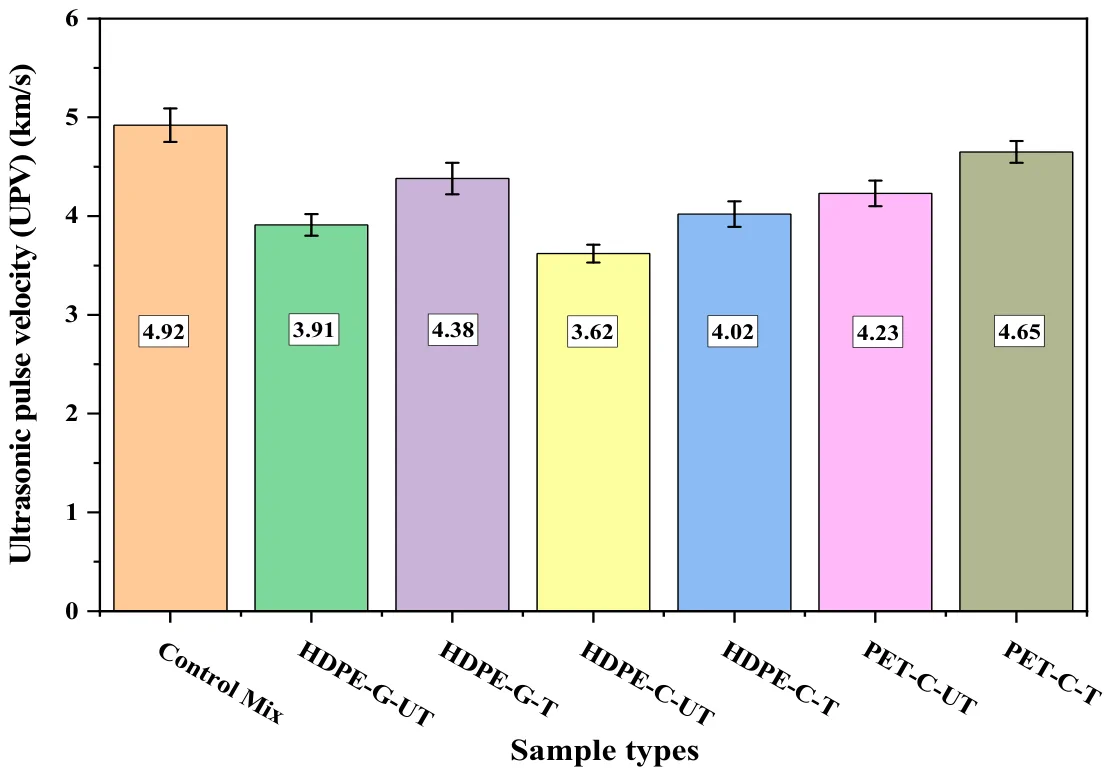
Ultrasonic pulse velocity (UPV) of control and recycled plastic aggregate concrete mixtures at 28 days of curing.

**Figure 12:**
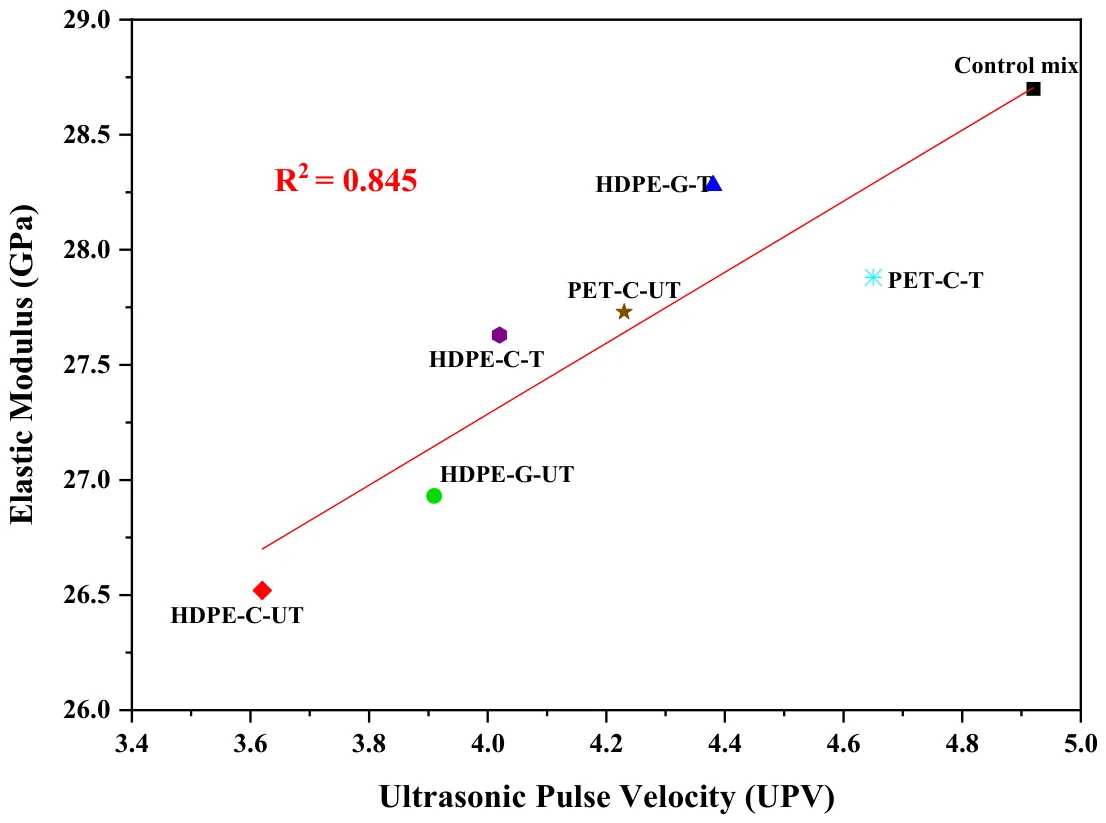
Correlation between ultrasonic pulse velocity and static elastic modulus.

Figure 11 presents the 28-day UPV of the concrete mixtures investigated. Incorporating untreated recycled PA reduced UPV relative to the control, indicating reduced internal continuity in the concrete. Among the untreated mixtures, HDPE-C-UT exhibited the lowest UPV, whereas HDPE-G-UT retained a comparatively higher wave velocity, suggesting that particle morphology influenced wave propagation through the concrete. Surface treatment significantly increased UPV across all recycled plastic mixtures. Relative to their untreated counterparts, the mean UPV increased by approximately 12.0% for HDPE-G, 11.0% for HDPE-C, and 9.9% for PET-C. These increases were statistically significant, with *p* = 0.024 for HDPE-G, *p* = 0.022 for HDPE-C, and *p* = 0.023 for PET-C. The consistent increase in UPV following treatment indicates improved ultrasonic wave transmission and is consistent with reduced internal discontinuities associated with recycled plastic incorporation. The UPV results generally followed the same trend as the elastic modulus and compressive strength. They also corresponded to the lower permeable void contents observed for the treated mixtures, suggesting that improved internal continuity contributed to the observed changes in stiffness and mechanical performance. To further examine the relationship between concrete stiffness and internal integrity, Figure 12 presents the correlation between the measured static elastic modulus and UPV. A strong positive correlation with r = 0.92 was observed, indicating that mixtures with higher UPV generally exhibited higher static elastic modulus. This correlation falls well above the 0.5 threshold associated with strong correlation in concrete-related Pearson correlation analysis [54]. The higher UPV values following surface treatment are consistent with reduced interfacial discontinuities and improved continuity of the plastic–cement ITZ, which may facilitate ultrasonic wave propagation and stress transfer across the interface. The observed relationship between UPV and static elastic modulus therefore supports the influence of interfacial quality on both wave propagation and composite stiffness.

### 4.4. FTIR and SEM–EDS Characterization

**Figure 13:**
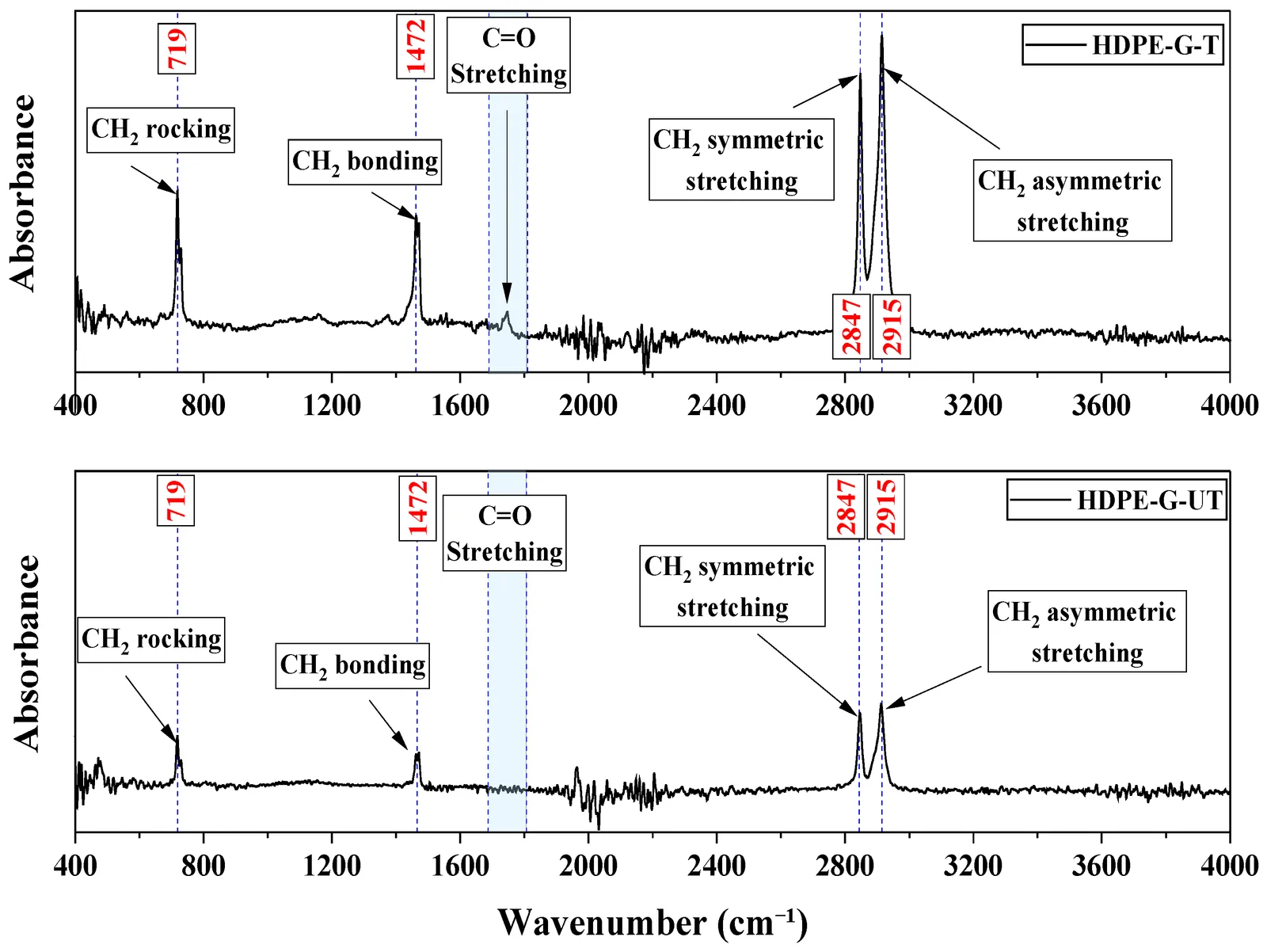
FTIR spectra of untreated and H_2_O_2_-treated HDPE-G.

FTIR spectra of untreated and treated HDPE-G are presented in Figure 13. The characteristic polyethylene bands near 719, 1472, 2847, and 2915 cm^−1^, corresponding to CH_2_ rocking, CH_2_ bending, symmetric C–H stretching, and asymmetric C–H stretching, respectively, were retained after H_2_O_2_ treatment. This indicates that the characteristic HDPE structure remained largely preserved. The principal treatment-associated difference was observed in the carbonyl region around 1700–1800 cm^−1^, where the treated HDPE-G exhibited a weak feature that was less evident in the untreated material. This change consists of limited oxidative modification of the HDPE surface rather than extensive degradation of the polymer backbone.

**Figure 14:**
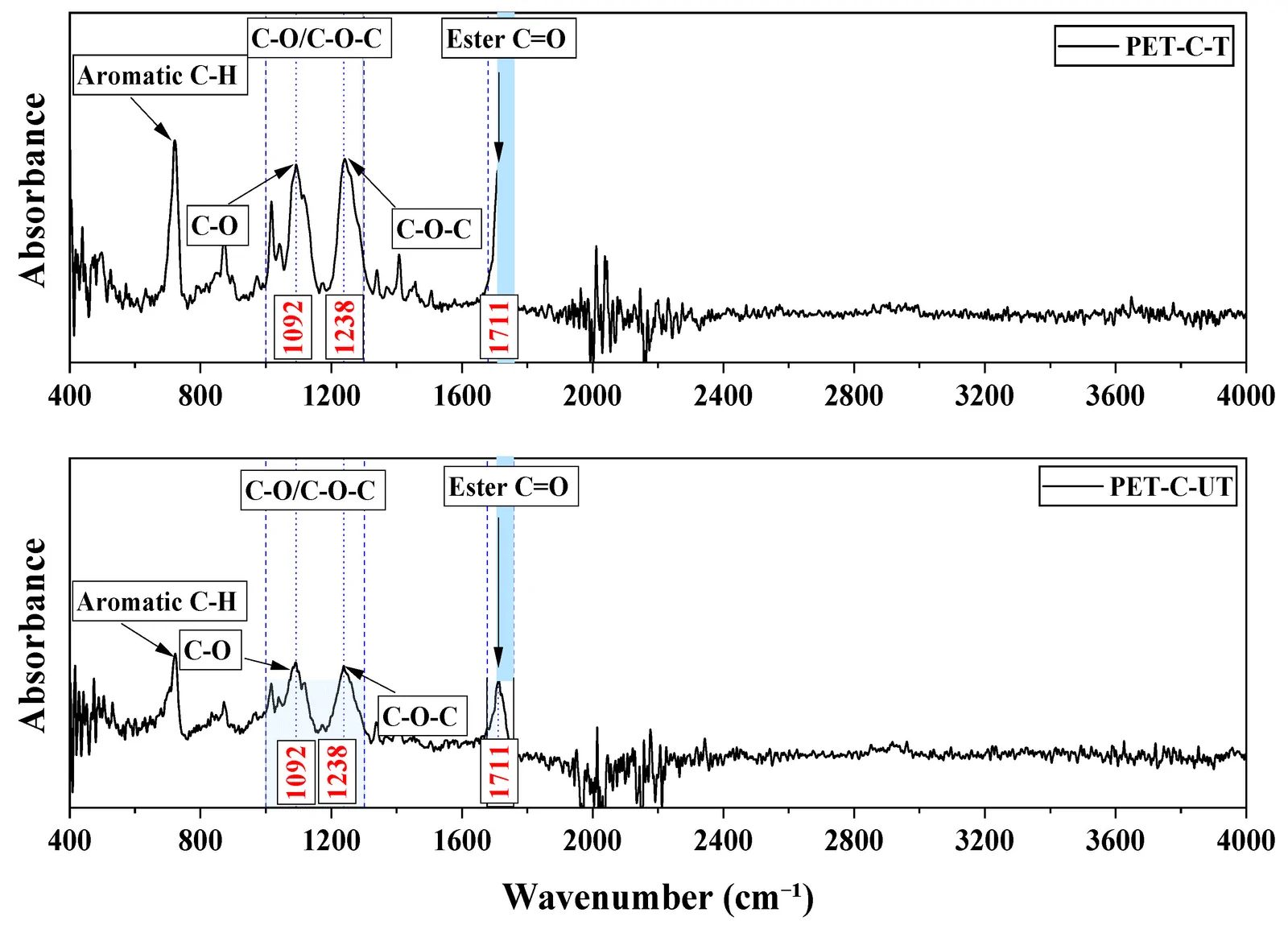
FTIR spectra of untreated and NaOH-treated PET-C.

FTIR spectra of untreated and NaOH-treated PET-C are shown in Figure 14. Both spectra retained the characteristic PET bands, including the ester C=O stretching band near 1711 cm^−1^ and C–O/C–O–C-related bands near 1092 and 1238 cm^−1^. Treatment-associated differences in these ester-related regions are consistent with modification of susceptible PET ester linkages during alkaline treatment. NaOH treatment of PET has similarly been associated with changes in ester and hydroxyl-related functionalities resulting from alkaline hydrolysis [55]. Together with the measured 3.58% mass loss, the present spectral changes support limited surface hydrolysis rather than extensive bulk degradation. The susceptibility of PET to alkaline interaction under cementitious environments has also been demonstrated using FTIR and SEM [56].

**Figure 15:**
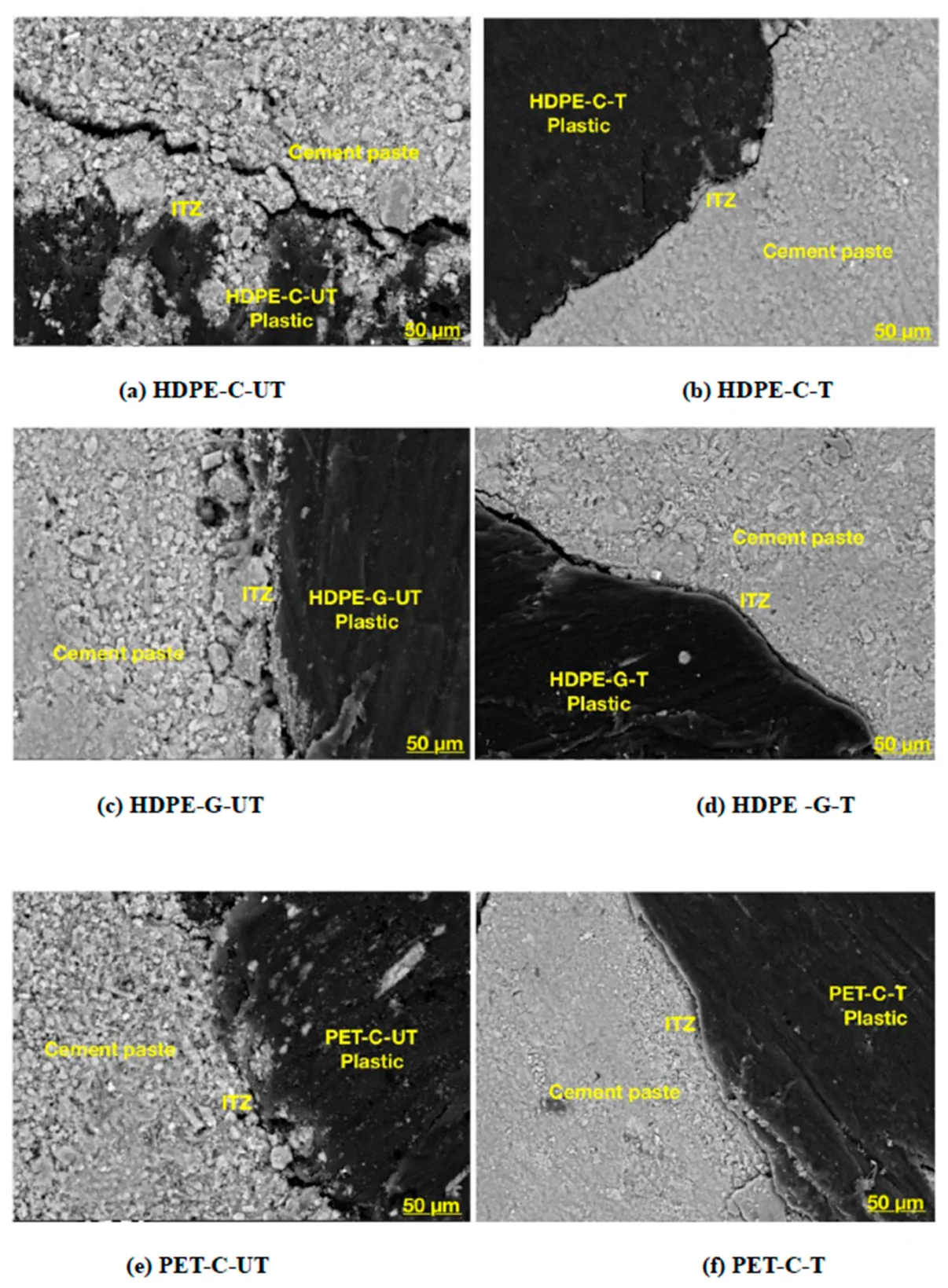
SEM micrographs of the plastic–cement paste interface.

SEM observations provided complementary evidence of treatment-associated changes at the plastic–cement paste interface (Figure 15). Untreated plastic concrete specimens generally exhibited localized gaps, interfacial separation, and discontinuous contact between the plastic particles and cementitious matrix. In contrast, the corresponding treated specimens showed closer and more continuous plastic–paste contact, although localized defects remained. Similar treatment-induced improvements in plastic–cement interaction have been reported by Abu-Saleem and Zhuge [57]. For PET, alkaline treatment has also been associated with increased surface roughness, which can promote mechanical interaction with the surrounding matrix [56].

EDS analysis further indicated treatment-associated variations in local interfacial composition. The Ca/Si ratio increased from 1.87 to 2.36 for HDPE-C and from 1.69 to 2.08 for PET-C, whereas HDPE-G decreased from 7.67 to 4.48. The Al/Si ratio showed no consistent treatment-dependent trend. The comparatively high Ca/Si values for HDPE-G likely reflect local sampling of Ca-rich or heterogeneous hydration products and should not be interpreted as direct evidence of superior interfacial bonding. Because EDS-derived elemental ratios depend on the locally sampled hydration phases, they are interpreted as semi-quantitative indicators of interfacial composition rather than direct measures of ITZ quality [58] (Table 6).

Overall, the FTIR and SEM–EDS results indicate that the treatments produced chemical and morphological modifications while largely preserving the characteristic polymer structures. The H_2_O_2_-treated HDPE exhibited evidence consistent with limited surface oxidation, whereas the NaOH-treated PET exhibited changes consistent with limited surface hydrolysis. These modifications, together with the reduced apparent interfacial separation observed by SEM, provide a plausible mechanism for the improved plastic–paste interaction and corresponding mechanical performance of the treated mixtures. Because SEM–EDS provides localized observations, these findings are interpreted as evidence of improved interfacial interaction rather than quantitative confirmation of ITZ densification.

### 4.5. Rapid Chloride Penetrability

**Figure 16:**
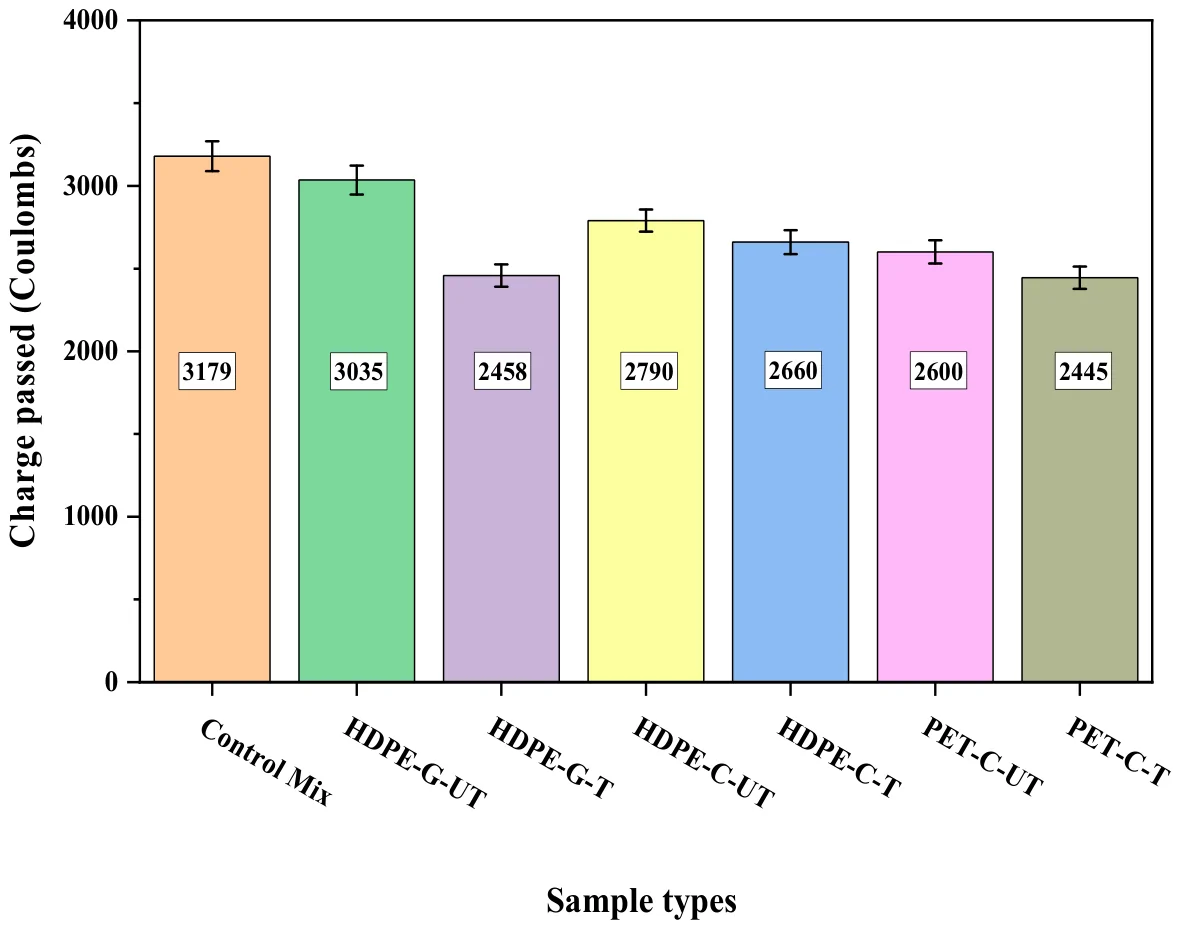
Rapid chloride penetrability of control and recycled plastic aggregate concrete mixtures.

Figure 16 presents the 28-day rapid chloride penetrability test results for the control and recycled plastic aggregate mixtures. Incorporating untreated recycled PA generally reduced the charge passed relative to the control, with HDPE-G-T and PET-C-T showing the lowest values across all mixtures. Surface treatment further reduced the mean charge passed for all recycled plastic aggregate types, although the statistical significance depended on polymer type. The reduction was statistically significant for HDPE-G, with *p* = 0.002, whereas the differences for HDPE-C, with *p* = 0.107, and PET-C, with *p* = 0.070, were not statistically significant. Therefore, the treatment effect on charge passed was statistically supported for HDPE-G, while the reductions observed for HDPE-C and PET-C are interpreted as numerical trends without statistical confirmation. The lower charge passed by the treated mixtures is consistent with reduced connectivity of permeable pores rather than simply a reduction in total porosity. Although recycled plastic particles are themselves impermeable, untreated mixtures may contain discontinuities around the particle surfaces that facilitate ionic transport. Surface treatment may reduce these discontinuities, resulting in a less continuous transport network and, consequently, lower charge passed. The electrical resistivity, water absorption, and permeable void results provide complementary evidence for this interpretation, although the statistical significance of the treatment effects varied among the polymer types. Together, these results suggest that polymer-specific surface treatment can improve resistance to ionic transport by reducing pore connectivity, with the strongest statistical evidence observed for HDPE-G. This behavior is consistent with percolation-based descriptions of ionic transport in cementitious composites, where transport resistance depends on whether connected pore pathways exceed a critical connectivity threshold rather than on total porosity alone [59].

### 4.6. Surface Electrical Resistivity

**Figure 17:**
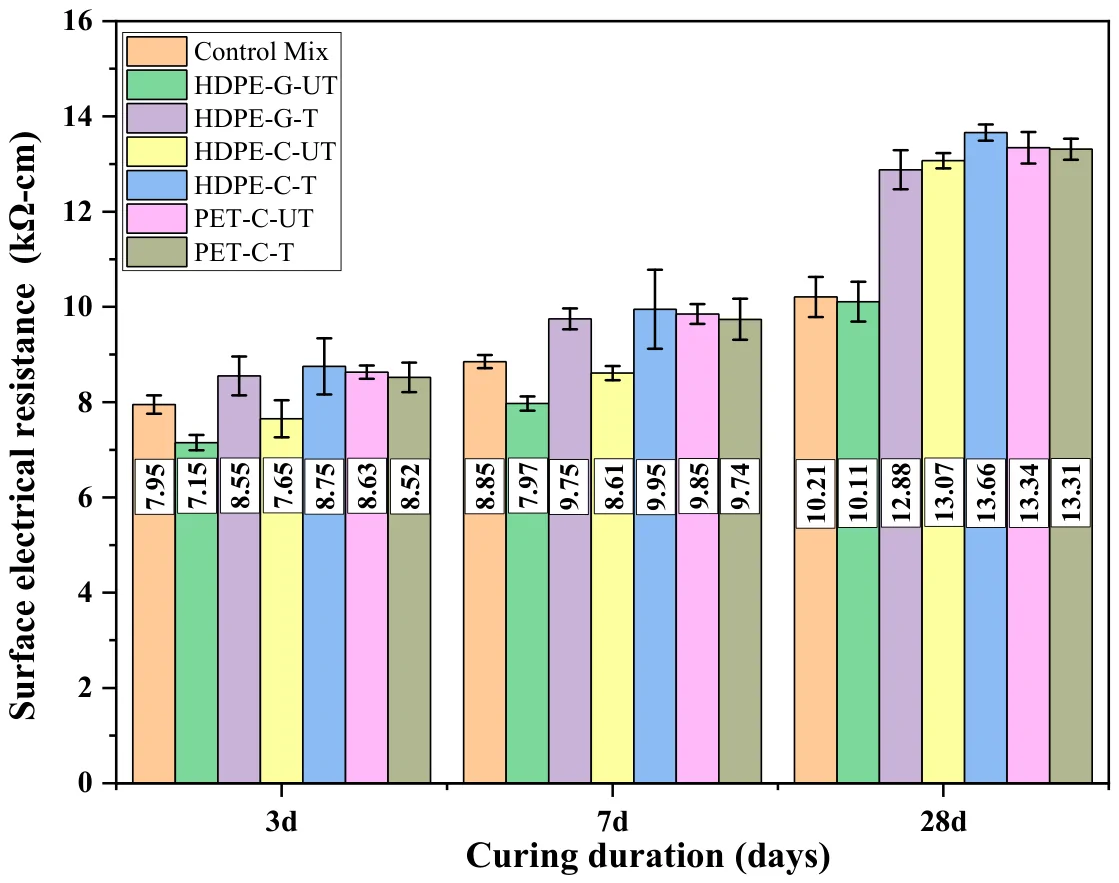
Surface electrical resistivity of control and recycled plastic aggregate concrete mixtures at 3,7 and 28 days of curing.

Figure 17 shows the surface electrical resistivity of the concrete mixtures investigated at 3, 7, and 28 days. Electrical resistivity increased with curing age for all mixtures as cement hydration progressively refined the pore structure. At 28 days, surface treatment significantly increased the electrical resistivity of both HDPE mixtures, with *p* = 0.003 for HDPE-G and *p* = 0.022 for HDPE-C. In contrast, the difference between untreated and treated PET-C was not statistically significant, with *p* = 0.904, indicating that NaOH treatment did not produce a statistically detectable change in its 28-day resistivity. The benefit of surface treatment on electrical resistivity was therefore polymer-dependent rather than universal. The higher resistivity of the treated HDPE mixtures is consistent with reduced continuity of moisture- and ion-conducting pathways within the concrete. A similar general trend was observed in the RCPT results, where treatment reduced the mean charge passed, although the statistical significance of the RCPT response also depended on polymer type.

**Figure 18:**
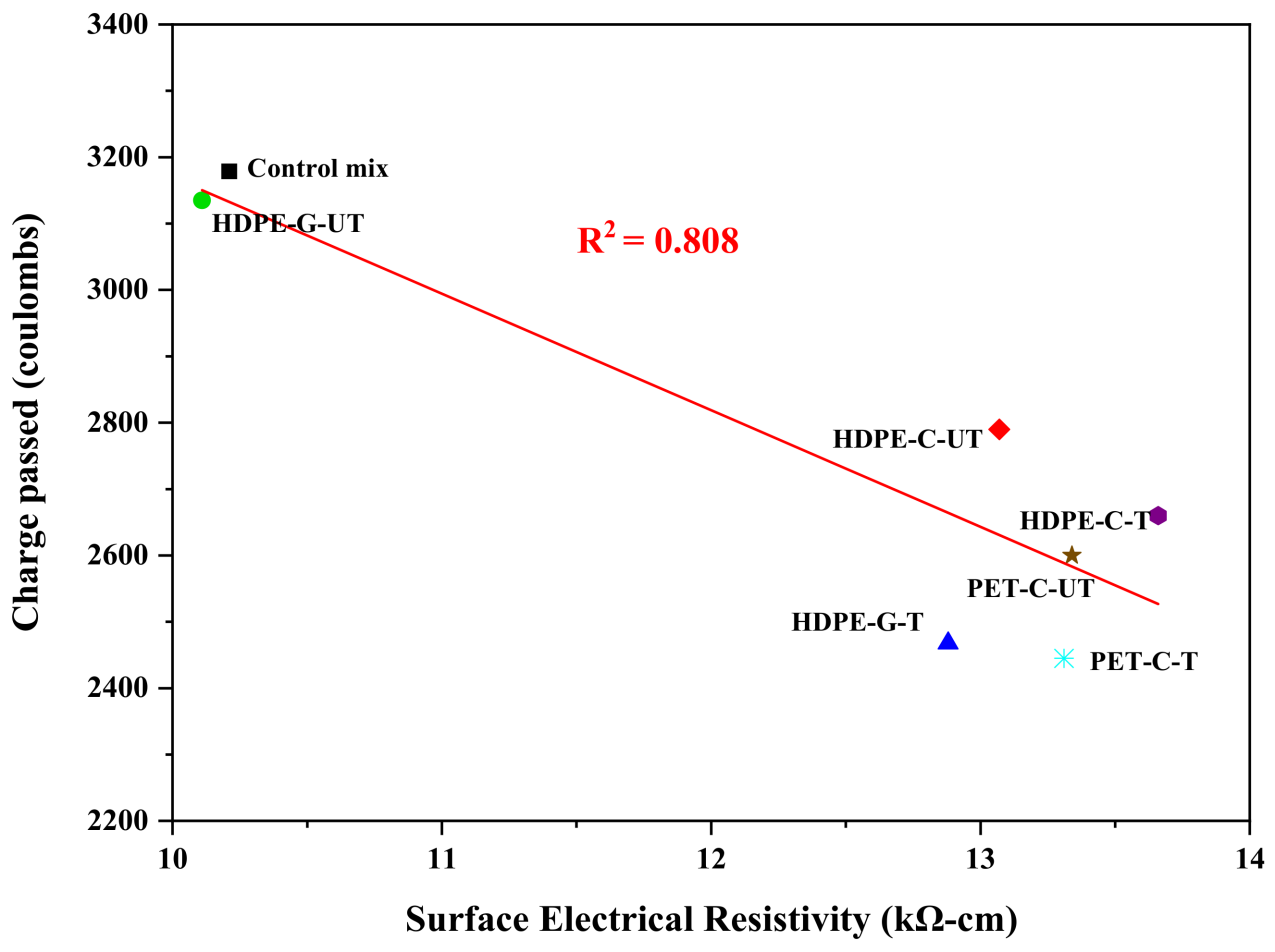
Correlation between surface electrical resistivity and charge passed during the RCPT.

Figure 18 shows the correlation between surface electrical resistivity and RCPT to further examine this relationship. A strong inverse correlation emerged, with R-squared equal to 0.808, showing that mixtures with higher resistivity generally passed less charge. This correlation falls well above the 0.5 threshold associated with strong correlation in concrete-related Pearson correlation analysis [54]. Resistivity reflects resistance to ionic conduction, while RCPT measures the electrical charge transported by the conductive pore solution under an applied voltage. Reducing the connectivity of interfacial defects and pore networks may therefore increase resistance to ionic movement while simultaneously reducing charge passed. PET-C is a partial exception to this relationship. Despite showing no statistically significant resistivity change following treatment, PET-C-T exhibited a lower RCPT charge passed than PET-C-UT, although this RCPT difference was also not statistically significant. This indicates that resistivity and RCPT, while strongly correlated overall, do not respond identically to surface treatment across all polymer types. PET’s inherently more polar surface chemistry, arising from the ester carbonyl groups in its polyester backbone, may afford PET-C a more compatible interface with the cement paste, even without surface treatment, than the non-polar hydrocarbon backbone of HDPE. The differing responses of resistivity and RCPT also reflect the different sensitivities of the two tests. Surface resistivity is strongly influenced by pore network connectivity and pore solution conductivity, while RCPT reflects electrical charge transport under an applied voltage and is additionally influenced by ionic mobility and Joule heating during the six-hour test. Documented cases of RCPT and surface resistivity producing different permeability classifications for the same concrete are reported in the literature, supporting their interpretation as complementary rather than interchangeable indicators of transport resistance [60].

### 4.7. Water Absorption and Permeable Voids

**Figure 19:**
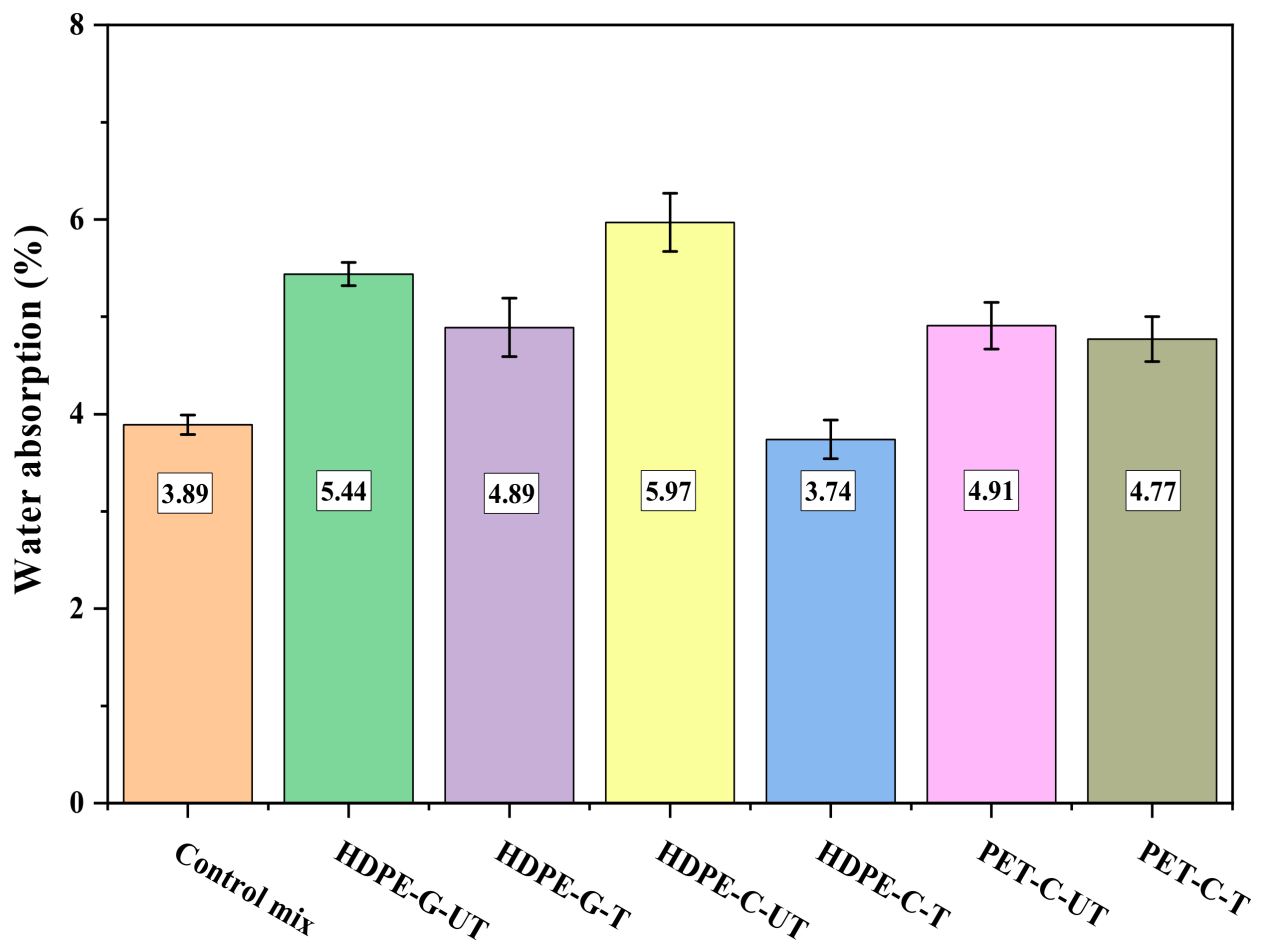
Water absorption (28-day).

**Figure 20:**
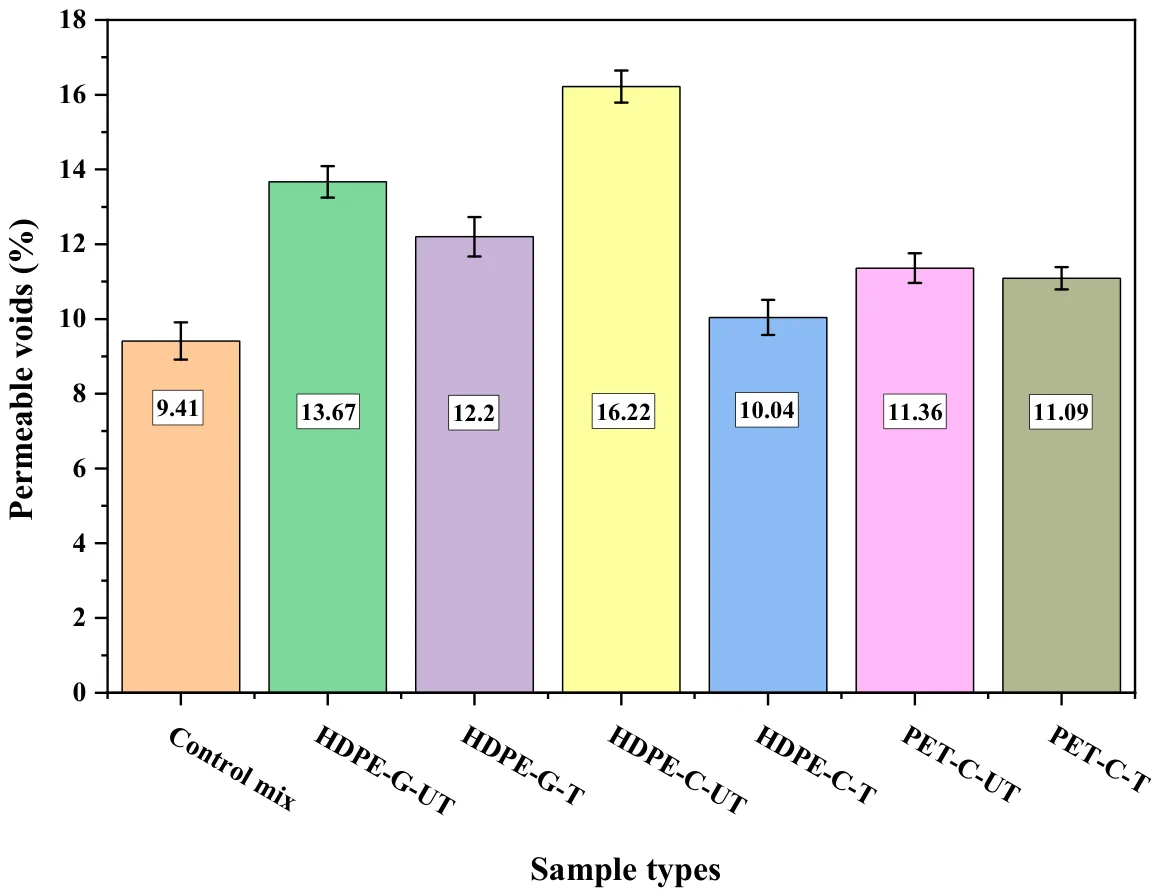
Permeable voids (28-day).

Figure 19 and Figure 20 present the 28-day water absorption and permeable void content of the investigated concrete mixtures. Incorporating untreated recycled plastic aggregates generally increased both water absorption and permeable void content relative to the control, indicating greater connectivity of moisture-accessible pores. Water absorption is governed primarily by capillary suction through interconnected pore networks rather than by isolated pores. Therefore, the higher values for the untreated mixtures suggest that weak plastic–cement ITZs created preferential pathways for moisture ingress. The HDPE-C-UT mixture exhibited the highest water absorption and permeable void content, indicating that particle morphology also influenced the development of interconnected transport pathways. Surface treatment produced numerical reductions in both water absorption and permeable void content across all recycled plastic mixtures, although the statistical significance was polymer- and property-dependent. For water absorption, the reduction was statistically significant only for HDPE-C, with *p* = 0.001, whereas the differences for HDPE-G, with *p* = 0.098, and PET-C, with *p* = 0.051, were not statistically significant. For permeable void content, significant reductions were observed for HDPE-G, with *p* = 0.029, and HDPE-C, with *p* = 0.0004, while the reduction for PET-C was not statistically significant, with *p* = 0.418. The significant reductions observed for the HDPE mixtures, particularly HDPE-C, support the effectiveness of H_2_O_2_ treatment in reducing moisture-accessible pore connectivity. The lower mean water absorption and permeable void contents of the treated mixtures are generally consistent with the electrical resistivity and RCPT results, although the statistical significance of the treatment response varied among polymer types and transport-related properties. Similar reductions in transport-related properties following polymer surface treatment have been reported in previous studies and are generally attributed to improved ITZ quality and reduced capillary connectivity.

### 4.8. Freeze–Thaw Durability

**Figure 21:**
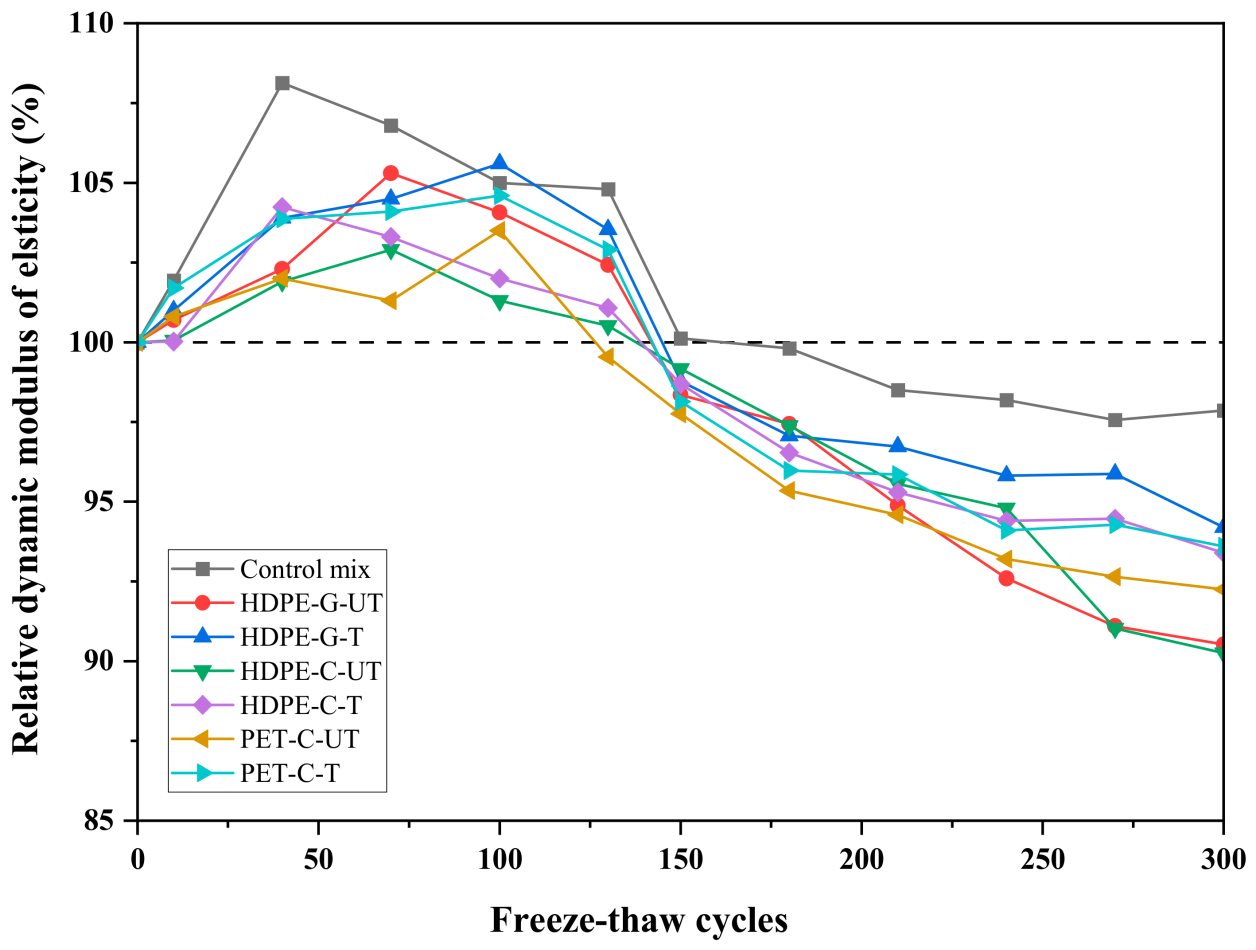
Relative dynamic modulus of elasticity.

**Figure 22:**
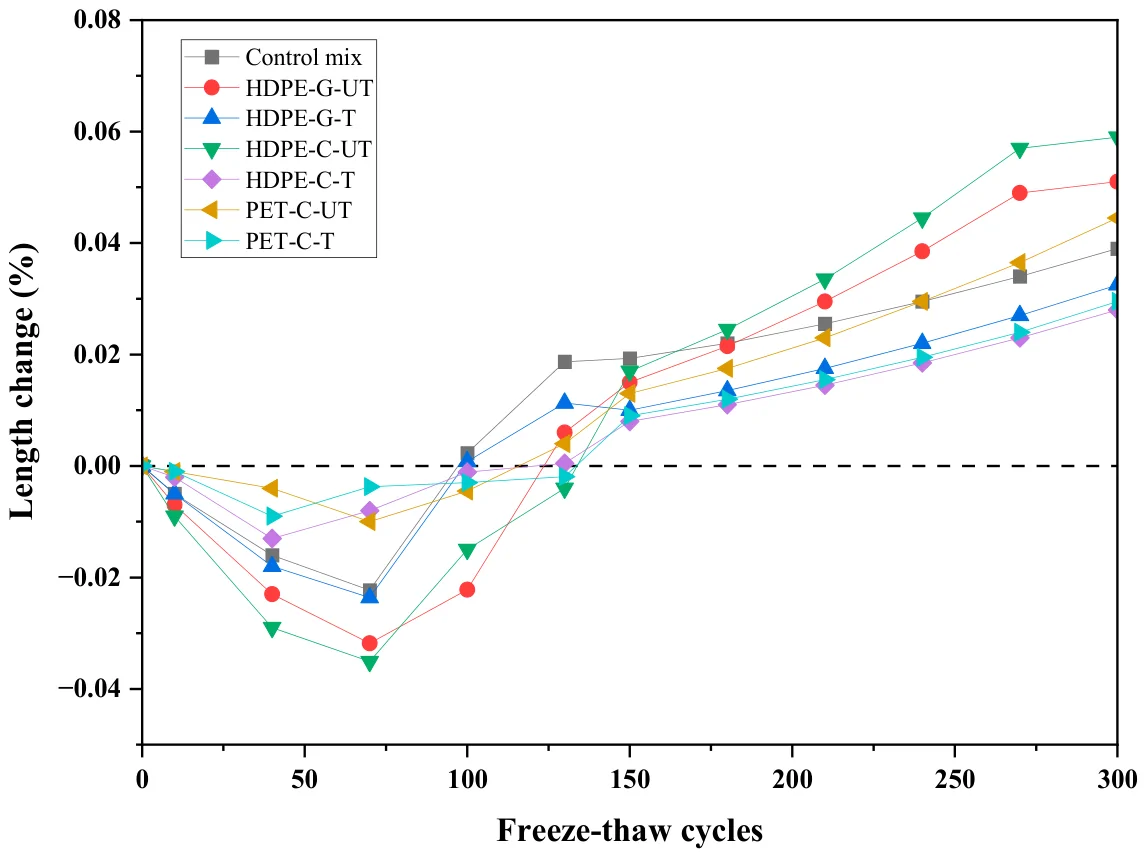
Length change.

**Figure 23:**
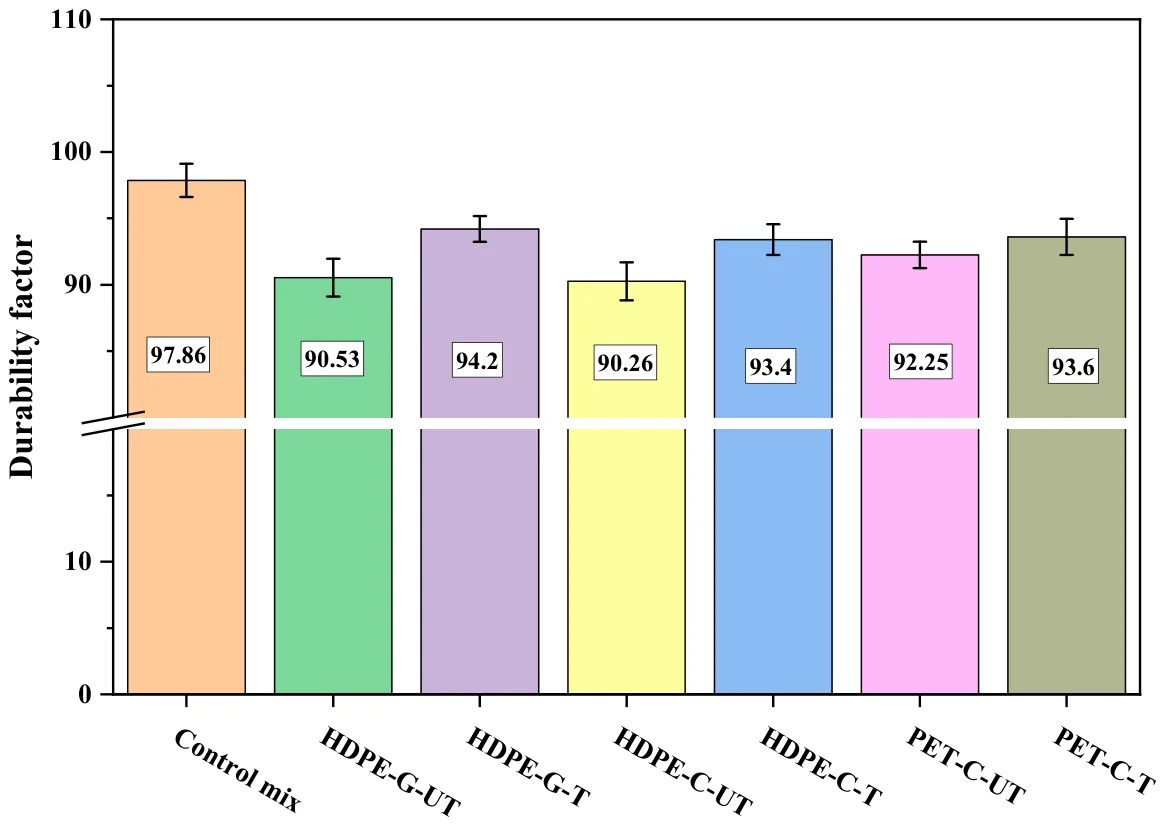
Durability factor.

Figure 21, Figure 22 and Figure 23 present the freeze–thaw performance of the investigated concrete mixtures in terms of relative dynamic modulus (RDM), length change, and durability factor. All mixtures exhibited a slight increase in RDM during the initial freeze–thaw cycles, followed by a gradual reduction with continued cycling. The initial increase likely reflects continued hydration of previously unhydrated cement particles, whereas the subsequent decline is attributed to the progressive development of frost-induced microcracks during repeated freezing and thawing [61,62]. No mixture fell below the 60% RDM failure threshold specified by ASTM C666 at any point through 300 cycles. Therefore, the durability factor for each mixture is equal to its RDM at the 300-cycle endpoint. All recycled plastic aggregate mixtures exhibited lower freeze–thaw durability than the control. Surface treatment increased the mean durability factor for all three plastic aggregate types, although the statistical significance depended on polymer type. The increases were statistically significant for HDPE-G, with *p* = 0.034, and HDPE-C, with *p* = 0.041, whereas the increase for PET-C was not statistically significant, with *p* = 0.258. Therefore, the treatment-related improvement in freeze–thaw durability was statistically supported for both HDPE mixtures, while the increase observed for PET-C represents a numerical trend without statistical confirmation. Across the six plastic aggregate mixtures, the durability factor decreased as permeable void content and water absorption increased, with coefficients of determination of R^2^ = 0.64 and R^2^ = 0.56, respectively, and increased strongly with 28-day compressive strength, with R^2^ = 0.90. These relationships indicate that moisture-accessible pore structure and matrix integrity govern the observed freeze–thaw response. This interpretation is consistent with Powers’ hydraulic pressure theory, in which freezing of confined pore water generates internal pressure that promotes microcracking when the pressure exceeds the tensile resistance of the surrounding cementitious matrix [61]. Recent extensions of this framework similarly account for cumulative hydraulic pressure effects during repeated freeze–thaw cycling [62].

Figure 22 further shows that the treated mixtures generally exhibited smaller length changes than their untreated counterparts, suggesting improved dimensional stability during cyclic freezing and thawing. This behavior is consistent with their generally lower water absorption and permeable void content and with improved ITZ characteristics, which may limit moisture ingress and frost-induced expansion [62].

### 4.9. Drying Shrinkage

**Figure 24:**
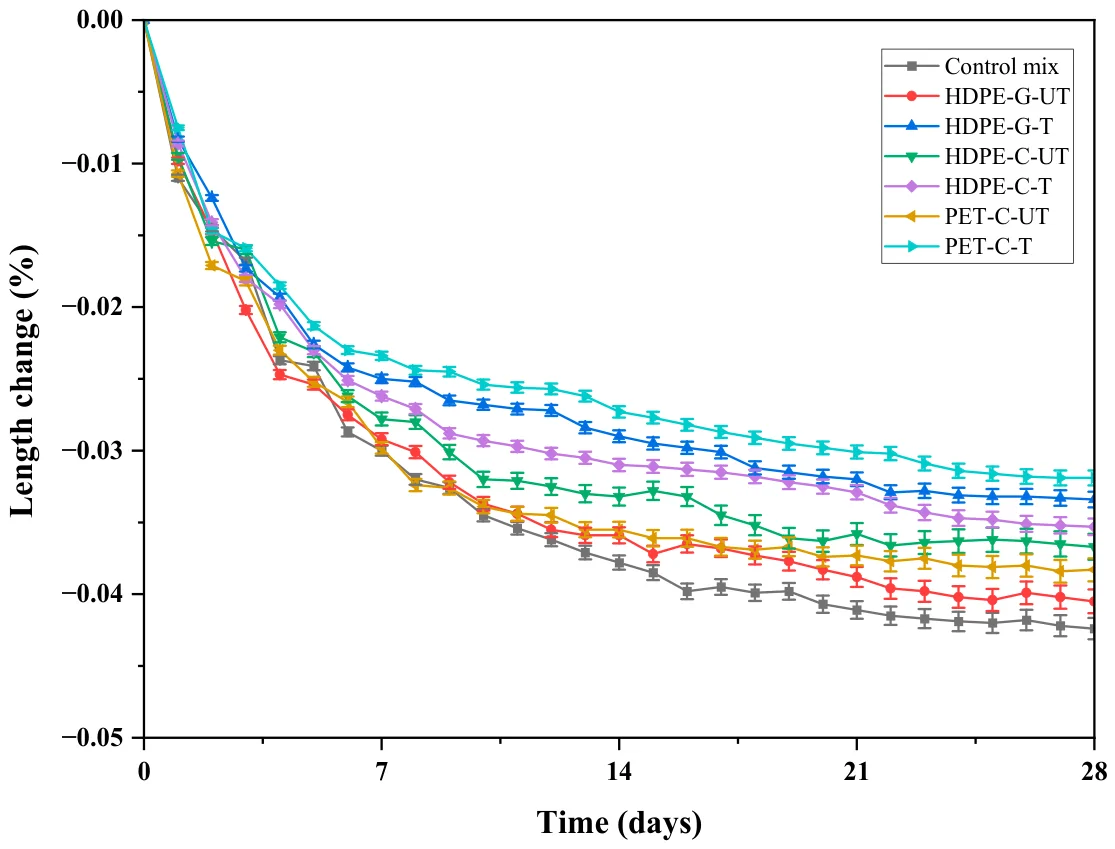
Drying shrinkage of control and recycled plastic aggregate concrete mixtures.

The drying shrinkage development shown in Figure 24 was investigated over 28 days. All mixtures exhibited rapid shrinkage during the first week, followed by a gradual reduction in shrinkage rate as drying time increased. Compared with the control, all recycled plastic mixtures exhibited lower drying shrinkage throughout the test period. Surface treatment further reduced the mean 28-day drying shrinkage of all recycled plastic mixtures, although the statistical significance depended on polymer type. The reductions were statistically significant for HDPE-G, with *p* = 0.0011, and PET-C, with *p* = 0.0013, whereas the difference for HDPE-C, with *p* = 0.1084, was not statistically significant. Therefore, the treatment-related reduction in drying shrinkage was statistically supported by HDPE-G and PET-C, while the reduction observed for HDPE-C represents a numerical trend without statistical confirmation. The lower shrinkage observed for the treated mixtures is consistent with changes in transport-related properties, including generally lower water absorption and permeable void content and higher electrical resistivity. These changes suggest reduced moisture transport through the concrete, which may contribute to the observed shrinkage response. The higher mean elastic modulus of the treated mixtures may also provide additional restraint against drying-induced deformation, although the statistical significance of the modulus response varied among polymer types.

**Figure 25:**
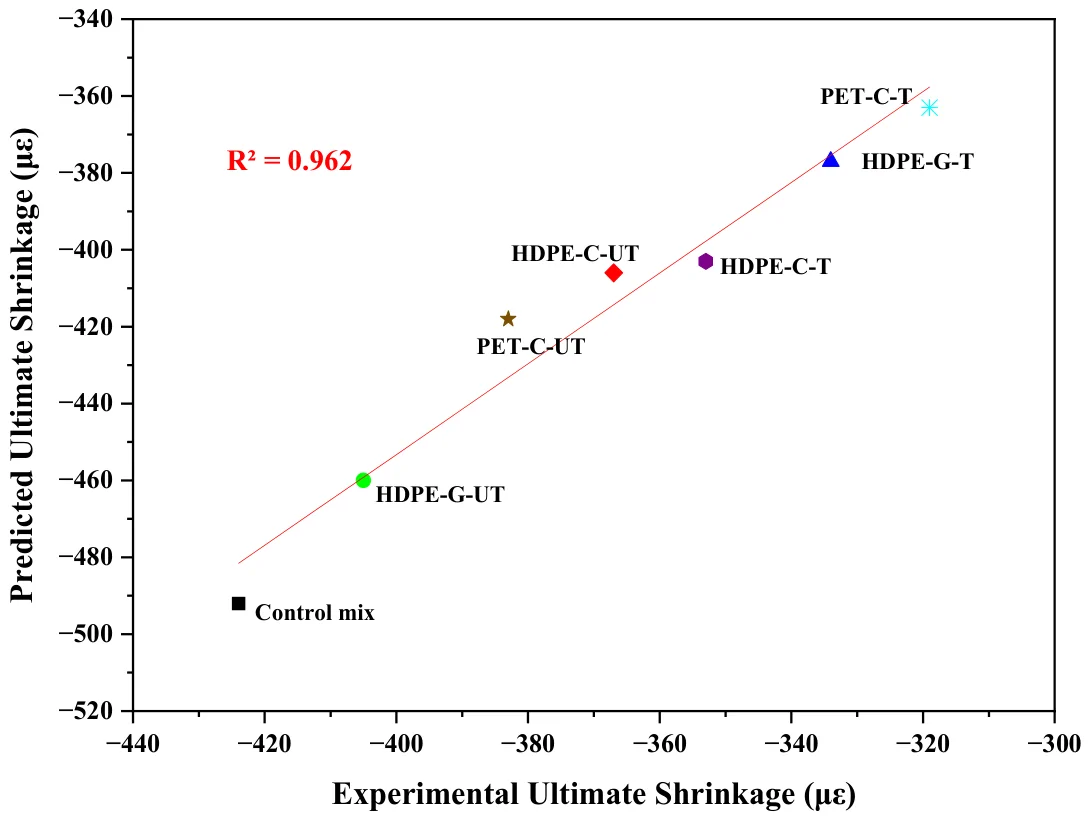
Comparison between experimental and predicted shrinkage (με).

The development of drying shrinkage was well described by Torben and Alan [63] and is shown in Figure 25. The hyperbolic model is

(3) $$\varepsilon \mathrm{sh}(t)=\varepsilon \mathrm{shu}\;\frac{t}{t+Ns}$$

where $\varepsilon_{sh}(t)$ is the drying shrinkage at time t, $\varepsilon_{shu}$ is the ultimate drying shrinkage, and $N_{s}$ is the time parameter corresponding to 50% of the ultimate shrinkage. The predicted shrinkage curves showed good agreement with the experimental measurements, indicating that the model adequately described the shrinkage evolution of the investigated mixtures. The lower $N_{s}$ values obtained for the PET mixtures suggest a more rapid development of early-age shrinkage than the corresponding HDPE mixtures.

### 4.10. Microscopic Analysis

**Figure 26:**
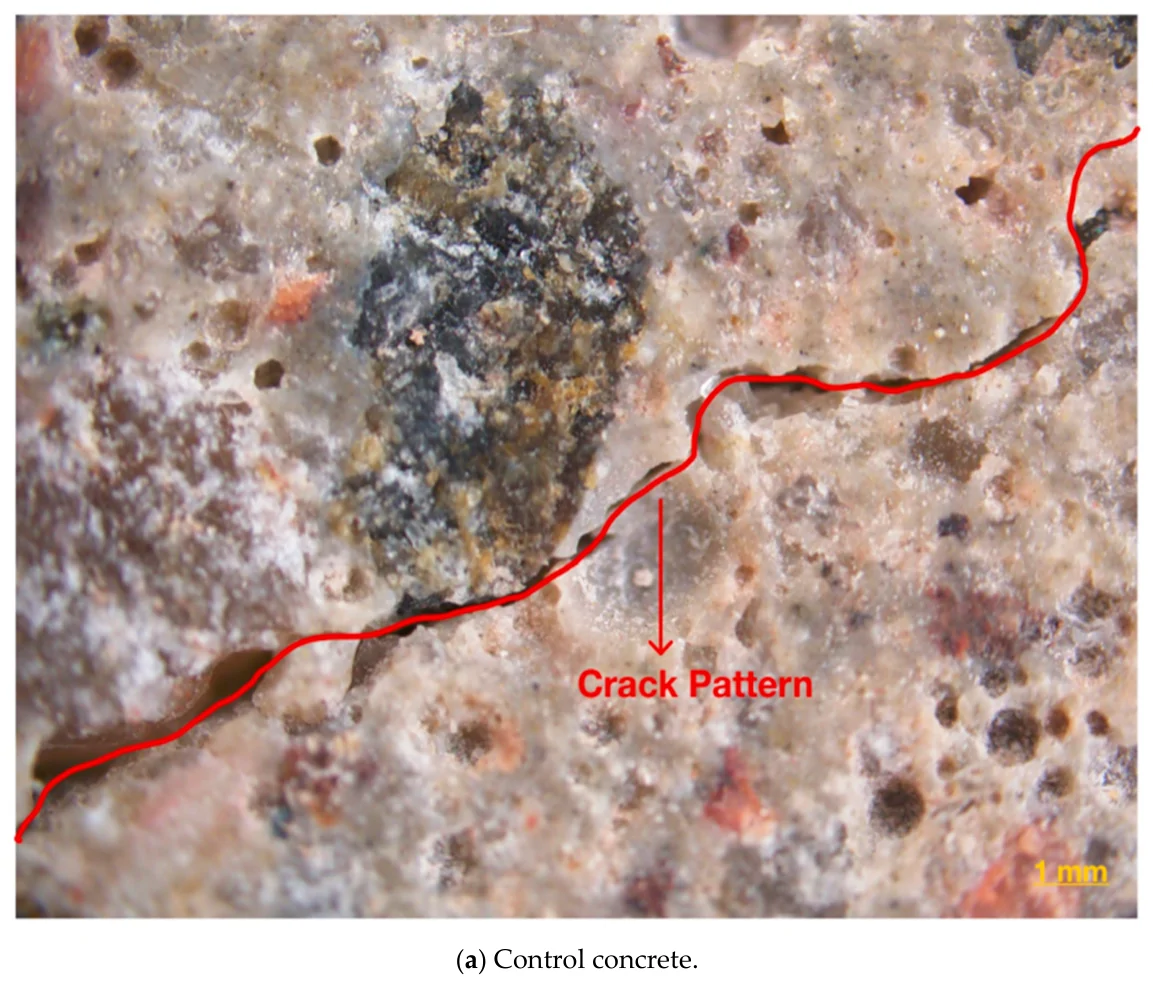

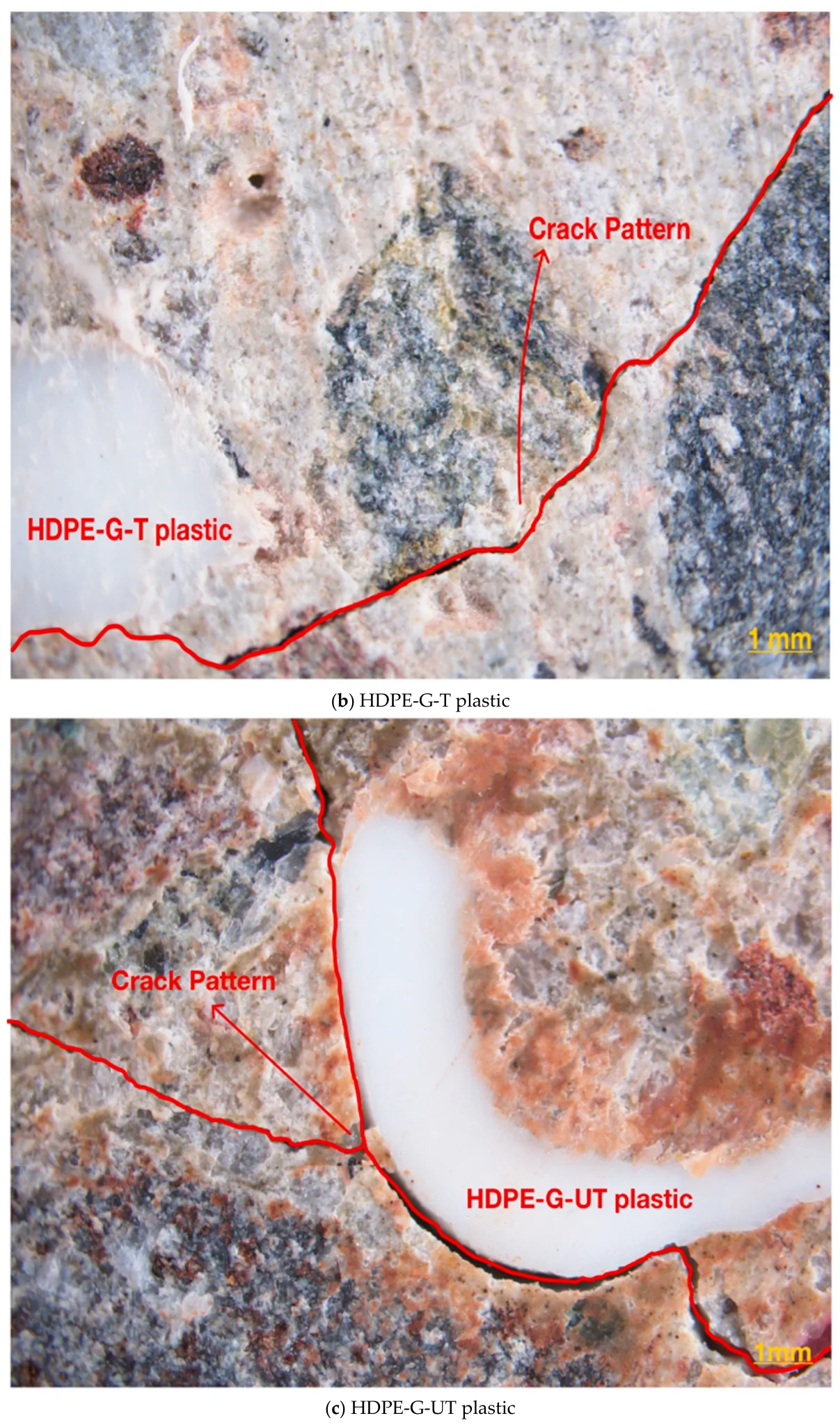

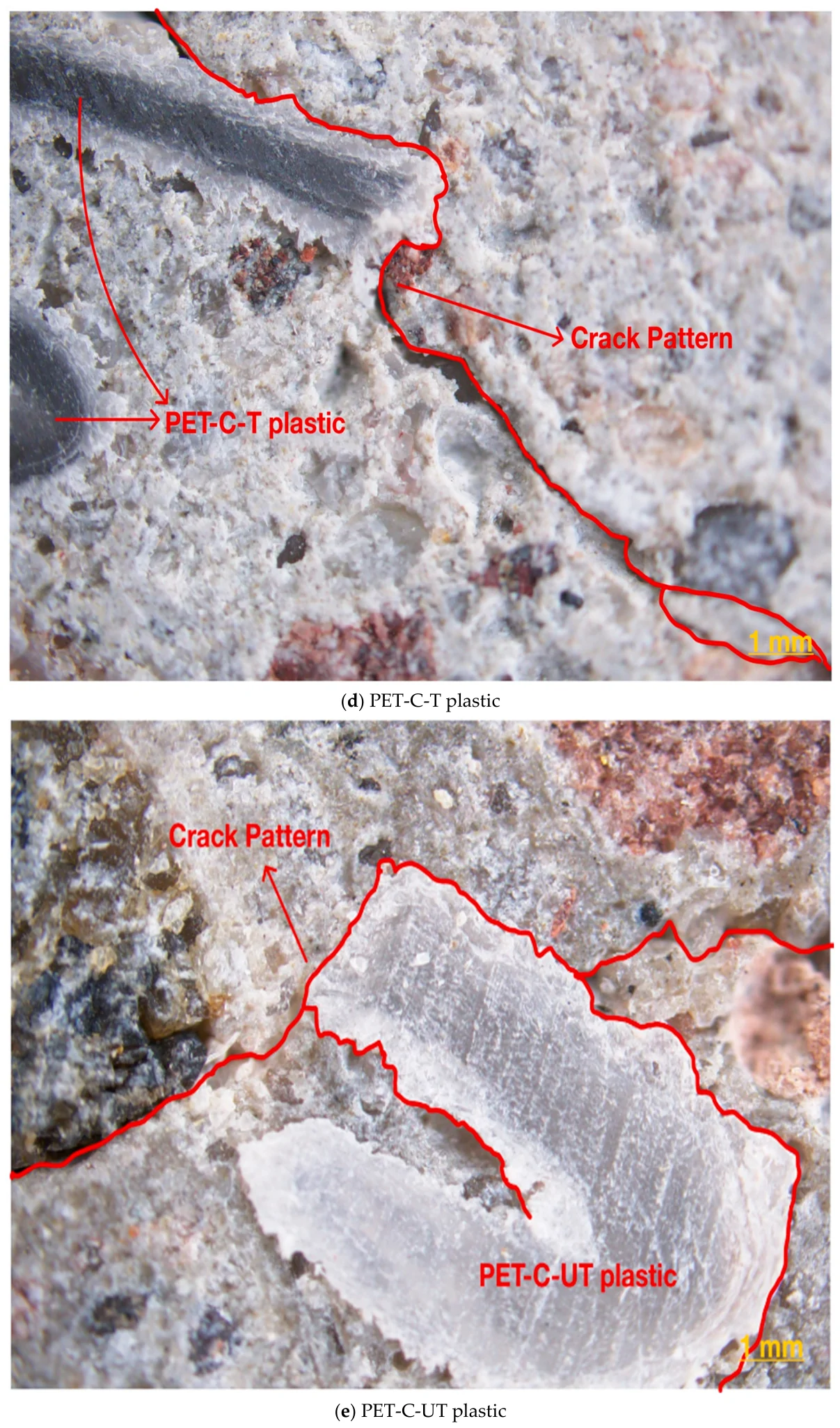

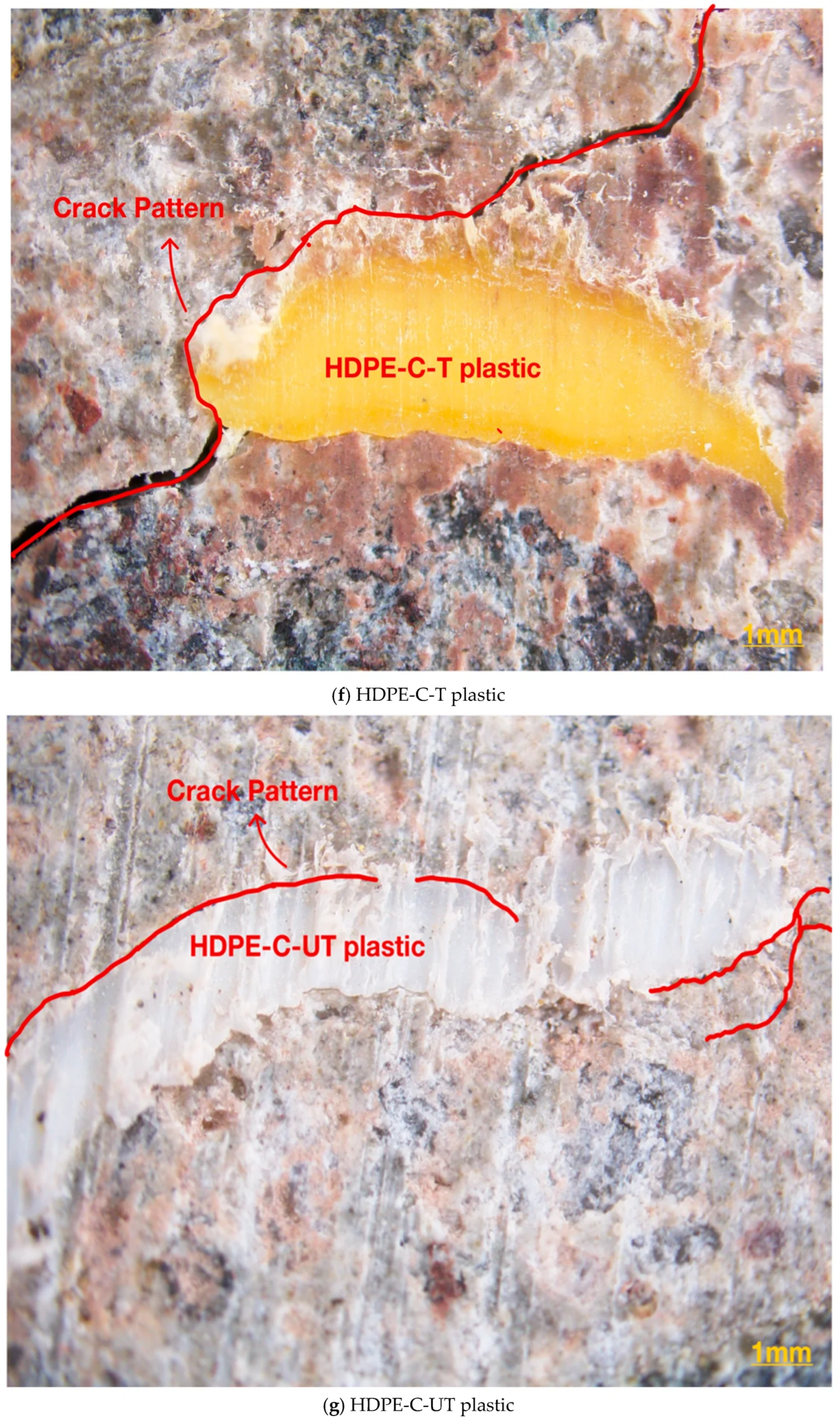
Crack propagation micrographs of control and recycled plastic aggregate concrete specimens after flexural loading.

Figure 26 presents crack propagation after flexural loading in the control and six plastic aggregate mixtures. All plastic aggregate specimens showed cracks following the plastic–cement interface rather than the surrounding matrix, reflecting the relatively large size of the plastic particles compared with the natural sand they replace, thereby extending the interfacial boundary along which a weak bond can develop, even after treatment. The control (Figure 26a) exhibited good aggregate–paste bonding, with the crack propagating through the cement matrix and natural aggregate ITZ rather than along a preferential weak interface, consistent with its highest flexural strength among all mixtures. HDPE-G-T (Figure 26b) and PET-C-T (Figure 26d) each showed a more continuous crack path along the particle boundary, whereas HDPE-G-UT (Figure 26c) and PET-C-UT (Figure 26e) exhibited visible interfacial gaps and more branched crack paths. Similarly, HDPE-C-T (Figure 26f) showed a more localized crack path, while HDPE-C-UT (Figure 26g) exhibited branches and multidirectional cracking along the interface.

The branched-versus-localized crack-path distinction was consistent across the three polymer types, with untreated specimens exhibiting greater crack branching and treated specimens showing more continuous propagation along the particle boundary. This behavior is consistent with the improved flexural strength of the treated mixtures and suggests that surface treatment reduced interfacial discontinuities and promoted more effective stress transfer. This interpretation is further supported by the SEM–EDS observations, which showed reduced apparent interfacial separation and more continuous plastic–paste contact in the treated specimens. FTIR characterization also demonstrated treatment-associated chemical modification of HDPE-G and PET-C, providing complementary evidence for the mechanisms underlying the observed interfacial response.

Although SEM–EDS provided direct microstructural observations and local compositional characterization of the plastic–paste interface, quantitative micromechanical characterization of ITZ thickness, hardness, indentation modulus, and bond strength was not performed. Nanoindentation mapping has been shown to quantify local variations in hardness and indentation modulus within interfacial regions of cementitious composites [64]. Therefore, the present SEM–EDS and crack-path observations are interpreted as evidence of improved interfacial interaction rather than quantitative confirmation of ITZ densification. Direct micromechanical characterization using nanoindentation and interfacial bond testing remains an important direction for future work.

## 5. Conclusions

This research evaluated how particle morphology and polymer-specific surface modification affected the fresh, mechanical, and durability characteristics of concrete incorporating 10% recycled plastic fine aggregate. The results indicate that incorporating recycled plastic aggregates generally reduced the mechanical and durability performance of concrete relative to the control mixture. However, the extent of these changes and the effectiveness of surface treatment were governed by the combined influence of particle morphology and polymer type rather than a uniform material response.

- All plastic aggregate mixtures increased the slump relative to the control, attributed to the non-absorptive polymer surface, which does not adsorb mixing water as natural sand does, thereby increasing the water available for flow. Because this is primarily a bulk rheological effect, surface treatment produced little change in slump.
- Untreated PA reduced compressive and tensile strength relative to the control, consistent with weaker plastic–paste interfacial interaction and less efficient stress transfer. Flexural behavior was additionally influenced by particle morphology, with the flat chip-shaped particles showing a different response under bending. Surface treatment produced numerical increases in the mechanical properties, but the statistical significance depended on the polymer type and loading mode. None of the treatment-related increases in 28-day compressive or splitting tensile strength were statistically significant. In contrast, the flexural strength increase of PET-C was statistically significant, while the increases for both HDPE mixtures were not. FTIR showed treatment-associated chemical modification while retaining the characteristic polymer structures, and SEM observations showed reduced apparent interfacial separation and more continuous plastic–paste contact in the treated specimens. These observations provide microstructural evidence consistent with improved interfacial interaction, although the resulting mechanical changes were not statistically significant for every property.
- Static elastic modulus and UPV decreased with untreated plastic incorporation, consistent with the lower intrinsic stiffness of the plastic aggregate and interfacial discontinuities within the composite. Surface treatment significantly increased the elastic modulus of HDPE-G, whereas the increases for HDPE-C and PET-C were not statistically significant. In contrast, UPV increased significantly following treatment for all three plastic aggregate types, supporting improved internal continuity following surface modification. H_2_O_2_-treated HDPE-G showed the closest modulus to the control, while the measured modulus of PET-C remained below the ACI 318 prediction even after treatment.
- The influence of surface treatment on transport-related properties was polymer-dependent. Over the 28 days, electrical resistivity increased significantly following treatment for both HDPE-G and HDPE-C, whereas no statistically significant change was observed for PET-C. The RCPT charge passed was significantly reduced for HDPE-G, while the reductions for HDPE-C and PET-C were not statistically significant. Water absorption was significantly reduced only for HDPE-C, while permeable void content was significantly reduced for HDPE-G and HDPE-C. Collectively, these results indicate that the most statistically consistent improvement in transport-related performance occurred for the H_2_O_2_-treated HDPE mixtures and is consistent with reduced connectivity of moisture- and ion-conducting pathways.
- All recycled plastic mixtures exhibited lower drying shrinkage than the control. Surface treatment further reduced the mean 28-day shrinkage of all plastic mixtures, with statistically significant reductions for HDPE-G and PET-C, whereas the reduction for HDPE-C was not statistically significant. The response was generally consistent with changes in transport-related properties and composite stiffness, although these effects varied among polymer types. The hyperbolic shrinkage model of Torben and Alan described the experimental data well, with predicted and experimental ultimate shrinkage correlating strongly, with R^2^ = 0.962.
- Incorporating plastic aggregate reduced freeze–thaw durability relative to the control, with durability factors for the recycled plastic mixtures ranging from 90.26% to 94.20% after 300 cycles. Surface treatment increased the mean durability factor for all three polymer types, with statistically significant increases for HDPE-G and HDPE-C, whereas the increase for PET-C was not statistically significant. The durability factor correlated negatively with permeable void content and water absorption and positively with 28-day compressive strength, supporting the influence of moisture-accessible pore structure and matrix integrity on freeze–thaw performance.

Overall, the findings of this study are limited to concrete with a 10% volumetric replacement of natural sand by recycled plastic aggregate, a water-to-cement ratio of 0.50, Type IL Portland cement without supplementary cementitious materials, and the curing and testing conditions adopted herein. Statistical analysis further demonstrates that numerical improvements following surface treatment should not be interpreted as uniform treatment effects, since statistical significance varies with polymer type and measured property. Higher replacement levels, alternative cementitious systems, and different curing regimes were not investigated and may alter the observed treatment effects. Although the results indicate potential for general concrete applications, the mixtures were not qualified for specific structural, precast, pavement, or aggressive chloride and freeze–thaw applications. ASTM C1202, ASTM C666, and AASHTO T358 provide standardized performance measures rather than application-specific acceptance criteria. Therefore, suitability should be established according to the governing structural and durability requirements for the intended service environment. Future studies should evaluate higher replacement levels, alternative cementitious systems and curing regimes, and long-term performance under different exposure conditions.

Several directions for future research follow from the limitations identified in this study. A size-matched mineral aggregate control would allow the contributions of gradation and packing to be separated more clearly from polymer-specific interfacial effects. Quantitative characterization of ITZ porosity and thickness, together with nanoindentation or direct interfacial bond measurements, would further establish the relationship between treatment-induced surface modification and local mechanical properties. Long-term studies under different curing regimes, cement compositions, replacement levels, and aggressive exposure conditions are also needed to establish application-specific performance limits.

## Figures and Tables

**Table 1:** Physical properties of all materials.

Property	Cement	Natural Sand	Coarse Aggregate	HDPE-G	HDPE-C	PET-C
Specific gravity (g/cm^3^)	3.14	2.71	2.65	0.95	0.95	1.39
Fineness modulus		2.71	—	4.86	4.90	4.76
Dominant sieve fraction (mm)	—	0.30–0.60	Max. 19	2.36–4.75	2.36–4.75	2.36–4.75
Particle morphology	Angular	Sub-angular	Angular (crushed)	Granular	Flat	Flat

**Table 2:** Particle size distribution (% passing) of natural sand and PA.

Sieve Size	Sand (% Passing)	HDPE-G (% Passing)	HDPE-C (% Passing)	PET-C (% Passing)	ASTM C33 [24] Limits (%)
4.75 mm	99.0	98.91	97.77	99.82	95–100
2.36 mm	91.0	13.87	11.35	21.93	80–100
1.18 mm	80.0	1.41	0.96	1.78	50–85
600 µm	46.0	0.19	0.00	0.17	25–60
300 µm	11.0	0.03	0.00	0.03	5–30
150 µm	2.0	0.00	0.00	0.00	0–10
Pan	0.0	0.00	0.00	0.00	—
Fineness modulus	2.71	4.86	4.90	4.76	2.3–3.1

**Table 3:** Simplified treatment chemical inventory and estimated production-stage carbon footprint per cubic meter of concrete.

Mixture	Treatment Agent	PA Content (kg/m^3^)	Bath L:S Ratio	Active Chemical Mass (kg)	Treatment Footprint (kg CO_2_e/m^3^)	Sand Footprint Avoided (kg CO_2_e/m^3^)	Ratio (Treatment: Avoided)
HDPE-G-T	20% H_2_O_2_	24	1.5:1	7.7	30.2	0.7	43:1
HDPE-C-T	20% H_2_O_2_	24	1.5:1	7.7	30.2	0.7	43:1
PET-C-T	20% NaOH	36	1.5:1	13.2	8.3	1.1	8:1

**Table 4:** Concrete mix designations.

Mix ID	PA	Treatment	Sand Replacement (vol%)
Control Mix	None (control)	None	0
HDPE-G-UT	HDPE-G	Untreated	10
HDPE-G-T	HDPE-G	20% H_2_O_2_	10
HDPE-C-UT	HDPE-C	Untreated	10
HDPE-C-T	HDPE-C	20% H_2_O_2_	10
PET-C-UT	PET-C	Untreated	10
PET-C-T	PET-C	20% NaOH	10

**Table 5:** Concrete mix proportions (kg/m^3^).

Mix ID	Cement	Water	Sand	PA	CA
Control Mix	380	190	670	0	1040
HDPE-G-UT	380	190	603	24	1040
HDPE-G-T	380	190	603	24	1040
HDPE-C-UT	380	190	603	24	1040
HDPE-C-T	380	190	603	24	1040
PET-C-UT	380	190	603	36	1040
PET-C-T	380	190	603	36	1040

**Table 6:** Local EDS-derived elemental ratios at the plastic–cement paste interface.

Sample	Ca/Si	Al/Si
HDPE-C-UT	1.87	0.23
HDPE-C-T	2.36	0.13
HDPE-G-UT	7.67	0.30
HDPE-G-T	4.48	0.25
PET-C-UT	1.69	0.09
PET-C-T	2.08	0.15

## Data Availability

The original contributions presented in this study are included in the article. Further inquiries can be directed to the corresponding author.

## Abbreviations

The following abbreviations appear in this manuscript:

CA	Coarse aggregate
DF	Durability factor
EDS	Energy-dispersive X-ray spectroscopy
FE-SEM	Field-emission scanning electron microscopy
FM	Fineness modulus
FTIR	Fourier-transform infrared spectroscopy
GWP	Global warming potential
ITZ	Interfacial transition zone
LCA	Life cycle assessment
MTS	Material testing system
PA	Plastic aggregate
PSD	Particle size distribution
RCPT	Rapid chloride penetrability test
RDM	Relative dynamic modulus
SCM	Supplementary cementitious material
SEM	Scanning electron microscopy
SSD	Saturated surface dry
T	Treated
UPV	Ultrasonic pulse velocity
UT	Untreated
w/c	Water-to-cement ratio

## References

[1] Plastics Europe (2025). Plastics The Fast Facts 2025: Global and European Plastics Production and Economic Indicators.

[2] Organisation for Economic Co-operation and Development (2022). Plastic Pollution is Growing Relentlessly as Waste Management and Recycling Fall Short, Says OECD.

[3] Pathak G.S., Hinge M., Otzen D.E. (2023). Transdisciplinary pragmatic melioration for the plastic life cycle: Why the social, natural, and technical sciences should prioritize reducing harm. Sci. Total Environ..

[4] Canopoli L., Fidalgo B., Coulon F., Wagland S.T. (2018). Physico-chemical properties of excavated plastic from landfill mining and current recycling routes. Waste Manag..

[5] Kibria M.G., Masuk N.I., Safayet R., Nguyen H.Q., Mourshed M. (2023). Plastic Waste: Challenges and Opportunities to Mitigate Pollution and Effective Management. Int. J. Environ. Res..

[6] Mia M., Karikar B.A., Mohibul S., Ali M.I., Khanam N., Siddiqui L. (2026). Environmental and Socio-economic Impacts of River Sand and Gravel Mining: A Review. Environ. Manag..

[7] Wattanavichien P., Iwanami M. (2024). Investigation of the mechanical, microstructure, and durability properties of concrete with fine uniform and non-uniform polyethylene terephthalate (PET) aggregates. Clean. Mater..

[8] Chen S.-J., Liu R.-A., Huang X.-Y., Chen Z.-B., Lin J.-X. (2024). Eco-friendly strain-hardening cementitious composites with recycled PET fine aggregate: Mechanical behavior and environmental benefits. J. Build. Eng..

[9] Silva R.V., de Brito J., Saikia N. (2013). Influence of curing conditions on the durability-related performance of concrete made with selected plastic waste aggregates. Cem. Concr. Compos..

[10] Saikia N., de Brito J. (2012). Use of plastic waste as aggregate in cement mortar and concrete preparation: A review. Constr. Build. Mater..

[11] Gu L., Ozbakkaloglu T. (2016). Use of recycled plastics in concrete: A critical review. Waste Manag..

[12] Frigione M. (2010). Recycling of PET bottles as fine aggregate in concrete. Waste Manag..

[13] Dawood A.O., Al-Khazraji H., Falih R.S. (2021). Physical and mechanical properties of concrete containing PET wastes as a partial replacement for fine aggregates. Case Stud. Constr. Mater..

[14] Basha S.I., Ali M.R., Shameem M., Al-Dulaijan S.U., Maslehuddin M. (2023). Durability of recycled waste plastic aggregate concrete. Eur. J. Environ. Civ. Eng..

[15] Qaidi S.M.A., Tayeh B.A., Zeyad A.M., de Azevedo A.R.G., Ahmed H.U., Emad W. (2022). Recycling of mine tailings for the geopolymers production: A systematic review. Case Stud. Constr. Mater..

[16] Ahmed A., Ahmad S., Musa A.E.S., Al-Osta M.A. (2024). Properties of concrete incorporating recycled coarse aggregates and recycled plastic fine aggregates. Innov. Infrastruct. Solut..

[17] Prasoon P.P., Kumar M.S.R. (2020). Durability Properties of Selfcompacting Concrete Incorporating Recycled High Density Polyethylene Plastics. Int. J. Adv. Res. Eng. Technol..

[18] Lee Z.H., Paul S.C., Kong S.Y., Susilawati S., Yang X. (2019). Modification of Waste Aggregate PET for Improving the Concrete Properties. Adv. Civ. Eng..

[19] Naguib H.M., Zaki E.G., Abdelsattar D.E., Dhmees A.S., Azab M.A., Elsaeed S.M., Kandil U.F. (2023). Environmentally Friendly Polymer Concrete: Polymer Treatment, Processing, and Investigating Carbon Footprint with Climate Change. ACS Omega.

[20] Abbas S.N., Qureshi M.I. (2025). Effect of recycled plastic aggregates on mechanical and durability properties of concrete: A review. Mater. Chem. Phys. Sustain. Energy.

[21] Nanda A., Panda S., Panigrahi S.K. (2025). Production of durable conventional concrete using recycled HDPE and PET plastic coarse aggregate. Innov. Infrastruct. Solut..

[22] Saikia N., de Brito J. (2014). Mechanical properties and abrasion behaviour of concrete containing shredded PET bottle waste as a partial substitution of natural aggregate. Constr. Build. Mater..

[23] (2023). Standard Specification for Blended Hydraulic Cements.

[24] (2023). Standard Specification for Concrete Aggregates.

[25] (2022). Standard Specification for Mixing Water Used in the Production of Hydraulic Cement Concrete.

[26] (2024). Entech Inc. https://4entech.com/.

[27] (2023). Standard Test Method for Sieve Analysis of Fine and Coarse Aggregates.

[28] Kasulanati M.L., Pancharathi R.K. (2024). Optimizing multi-recycled concrete for sustainability: Aggregate gradation, surface treatment methods and life cycle impact assessment. Constr. Build. Mater..

[29] Khanapur N.V., Tripathi B., Pradhan S., Chandra T. (2025). Synergistic effect of particle packing method, aggregate saturation levels, and paste content in improving the performance of high volume fine recycled aggregate concrete. Sci. Rep..

[30] Baten B., Garg N. (2024). Introducing Particle Shape Metric (PSM): A fundamental parameter that encapsulates role of aggregate shape in enhancing packing and performance. Cem. Concr. Res..

[31] Zhuo J., Zhang Y., Ma M., Zhang Y., Zheng Y. (2022). Uniaxial Compression Failure and Size Effect of Recycled Aggregate Concrete Based on Meso-Simulation Analysis. Materials.

[32] Anum I., Williams F.N., Zakka W.P., Keftin N.A. (2025). Effects of treated high-density polyethylene admixtures on the compressive strength of concrete in sulphates and acids environments. Discov. Civ. Eng..

[33] Abedsoltan H. (2023). A focused review on recycling and hydrolysis techniques of polyethylene terephthalate. Polym. Eng. Sci..

[34] Giraldo-Narcizo S., Guenani N., Sánchez-Pérez A.M., Guerrero A. (2023). Accelerated Polyethylene Terephthalate (PET) Enzymatic Degradation by Room Temperature Alkali Pre-treatment for Reduced Polymer Crystallinity. ChemBioChem.

[35] Coviello C.G., La Scala A., Sabbà M.F., Carnimeo L. (2024). On the Cementitious Mixtures Reinforced with Waste Polyethylene Terephthalate. Materials.

[36] Guo J., Li K., Liang J., Ying D., Song X., He Y., Jia J. (2026). High-quality hydrogen peroxide production through electrochemical reduction of anthraquinone in ambient environment. Sci. Adv..

[37] Thannimalay L., Yusoff S., Zin Zawawi N. (2013). Life Cycle Assessment of Sodium Hydroxide. Aust. J. Basic Appl. Sci..

[38] Sala D., Liashenko O., Pyzalski M., Pavlov K., Pavlova O.M., Durczak K., Chornyi R. (2025). The Energy Footprint in the EU: How CO2 Emission Reductions Drive Sustainable Development. Energies.

[39] (2023). Standard Test Method for Slump of Hydraulic-Cement Concrete.

[40] (2023). Standard Test Method for Density (Unit Weight), Yield, and Air Content (Gravimetric) of Concrete.

[41] (2023). Standard Test Method for Bulk Density (“Unit Weight”) and Voids in Aggregate.

[42] (2023). Standard Test Method for Compressive Strength of Cylindrical Concrete Specimens.

[43] (2022). Standard Test Method for Flexural Strength of Concrete (Using Simple Beam with Third-Point Loading).

[44] (2014). Standard Test Method for Static Modulus of Elasticity and Poisson’s Ratio of Concrete in Compression.

[45] (2016). Standard Test Method for Pulse Velocity Through Concrete.

[46] (2025). Standard Test Method for Electrical Indication of Concrete’s Ability to Resist Chloride Ion Penetration.

[47] (2019). Standard Method of Test for Surface Resistivity Indication of Concrete’s Ability to Resist Chloride Ion Penetration.

[48] (2021). Standard Test Method for Density, Absorption, and Voids in Hardened Concrete.

[49] (2026). Standard Test Method for Resistance of Concrete to Rapid Freezing and Thawing.

[50] (2024). Standard Test Method for Length Change of Hardened Hydraulic-Cement Mortar and Concrete.

[51] Cook M.D., Ley M.T. (2023). Impacts of Fine Aggregate Gradation on the Workability of Slip-Formed Concrete. J. Mater. Civ. Eng..

[52] Steven H., Kosmatka B.K., William C.P. (2002). Design and Control of Concrete Mixtures.

[53] Yan S., Wang B., Sun Y., Lyu B. (2021). Micromechanics-Based Prediction Models and Experimental Validation on Elastic Modulus of Recycled Aggregate Concrete. Sustainability.

[54] Mohamad H.M., Asman N.A., Mirasa A.K., Saad I., Bolong N., Steven C.C.K., Siti Nooraiin M.R. (2021). A Consistency Check of Concrete Compressive Strength using Pearson’s Correlation Coefficient. Civ. Eng. J..

[55] Ghamarpoor R., Jamshidi M., Sayyadian M., Razavizadeh M. (2023). Chemical/photochemical functionalization of polyethylene terephthalate fabric: Effects on mechanical properties and bonding to nitrile rubber. Sci. Rep..

[56] Silva D.A., Betioli A.M., Gleize P.J.P., Roman H.R., Gómez L.A., Ribeiro J.L.D. (2005). Degradation of recycled PET fibers in Portland cement-based materials. Cem. Concr. Res..

[57] Abu-Saleem M., Zhuge Y., Hassanli R., Ellis M., Rahman M.M., Levett P. (2021). Microwave radiation treatment to improve the strength of recycled plastic aggregate concrete. Case Stud. Constr. Mater..

[58] Qin X., Yin M., Yang T., Li X., Zhu Q., Gao X. (2025). Surface coating treatment of recycled concrete aggregate using recycled concrete powder-based geopolymer: Properties and interfacial transition zone. Constr. Build. Mater..

[59] Paswan R., Das S. (2026). Mechanistic links between multiscale pore network evolution and chloride transport in PCM-integrated concretes subjected to freeze–thaw cycling. Cem. Concr. Compos..

[60] Mousavinezhad S., Nozari S., Newtson C.M. (2025). Classifying Concrete Permeability Using Rapid Chloride Permeability and Surface Resistivity Tests: Benefits, Limitations, and Predictive Models—A State-of-the-Art Review. Buildings.

[61] Powers T.C. (1945). A Worlcing Hypothesis for Further Studies of Frost Resistance of Concrete. J. Am. Concr. Inst..

[62] Xu L., Wang Y., Wang Y., Cheng T. (2025). A New Concrete Freeze–Thaw Damage Model Based on Hydraulic Pressure Mechanism and Its Application. Materials.

[63] Torben C.H., Alan H.M. (1966). Influence of Size and Shape of Member on the Shrinkage and Creep of Concrete. ACI J. Proc..

[64] Huo W., Zhang S., Zhu Z., Sun H., Wan Y., Zhang C., Yang L. (2025). ITZs characterization in full-component geopolymer recycled concrete based on quantitative BSE-EDS images and nanoindentation techniques. Constr. Build. Mater..

